# The Dynamic Formation from Metal‐Organic Frameworks of High‐Density Platinum Single‐Atom Catalysts with Metal‐Metal Interactions

**DOI:** 10.1002/anie.202213412

**Published:** 2022-10-27

**Authors:** Jieqiong Shan, Jiangwen Liao, Chao Ye, Juncai Dong, Yao Zheng, Shi‐Zhang Qiao

**Affiliations:** ^1^ School of Chemical Engineering and Advanced Materials The University of Adelaide Adelaide SA 5005 Australia; ^2^ Beijing Synchrotron Radiation Facility Institute of High Energy Physics Chinese Academy of Sciences Beijing 100049 China

**Keywords:** Hard-Soft Acid-Base Principle, Metal-Metal Interactions, Metal-Organic Frameworks, Pyrolysis, Single Atom Catalysts

## Abstract

Single‐atom catalysts (SACs) hold great promise for highly efficient heterogeneous catalysis, yet the practical applications require the development of high‐density active sites with flexible geometric structures. The lack of understanding in the dynamic formation process of single atoms in the host framework has been plaguing the controllable synthesis of next generation SACs. Here using Co‐based metal‐organic frameworks (MOFs) as a starting substrate, we fully elucidated the formation of high‐density Pt single atoms with inter‐site interactions in derived Co_3_O_4_ host. The cation exchange process and dynamic evolution of Pt−Pt interactions, organic ligand cleavage and Pt‐oxygen coordination formation during the pyrolysis process have been unambiguously interpreted by a series of in situ/ex situ spectroscopic measurements and theoretical computation. These findings would direct the synthesis of high‐density SACs with metal‐metal interactions, which demonstrate significantly enhanced structural flexibility and catalytic properties.

## Graphical abstract

The incorporation of metal cations in metal‐organic frameworks (MOFs) via a cation exchange process is governed by the Hard‐Soft Acid‐Base (HSAB) principle. During pyrolysis, the evolution of first‐shell Pt‐ligand coordination determines the formation of the Pt−Pt second‐shell coordination, which promotes the immobilization of Pt atoms as high‐density single‐atom catalysts in the Co_3_O_4_ host.

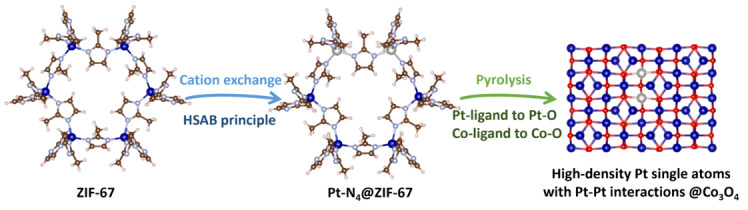

## Introduction

Single‐atom catalysts (SACs) have attracted extensive research interests over the past decade because of the maximum atomic utilization and unique geometric structure, which are significant for reducing cost and improving intrinsic activity of metal‐based catalysts.^[1]^ The preparation of SACs has been widely achieved on most transition metals and noble metals by the wet‐chemical approach.^[2]^ As an emerging platform for the construction of SACs, metal‐organic frameworks (MOFs) provide spatially separated metal sites, tailorable structures and flexible coordination for incorporated guest metal atoms.^[3]^ A strong metal‐support interaction between metal single atoms and substrate is often required to prevent aggregation of the former, which however always leads to low loadings (<2 weight %) of fully isolated single atoms.^[4]^ The low density of metal active sites limits the apparent catalytic activity of SACs. More importantly, it is recognized that the structural simplicity of fully isolated single atoms restricts the substantial electronic structure modulation, thus hinders the catalytic property optimization of SACs.^[5]^ For example, the isolated single atoms are generally in random and uncontrollable spatial distribution and lacks collective effects among active sites. On the other hand, the high‐density SACs are promising to achieve enhanced overall apparent catalytic performance towards practical applications.^[6]^ Especially, with increased density, adjacent single atoms exhibiting site‐to‐site interaction would provide unique adsorption configuration and electronic structure.^[7]^ This permits inter‐site synergy to break scaling relations of various intermediates and thus promote catalytic activity of SACs.^[8]^ However, high‐density SACs with specific correlation among single atoms are challenging to fabricate.^[9]^

Some efforts have been devoted to increasing atomic density of single atoms, e.g., developing diatomic/multiatomic metal pairs/clusters,^[10]^ triggering spatial correlation among metal single atoms,^[11]^ etc. These catalysts demonstrate synergistic effects via atomic metal‐metal interactions among neighboring single atoms and allow effective regulation of catalytic property meanwhile maintains remarkable atomic utilization of conventional SACs.^[7b]^ It is an efficient strategy to induce metal‐metal interaction among high‐density single atoms within MOFs.^[12]^ Generally, the fabrication of SACs starting from MOFs consists of two steps: the anchor of target single metal sites by cation exchange into the MOFs substrate and the removal of excess ligands by pyrolysis to ensure accessibility of metal sites.^[13]^ While this synthetic strategy has been widely applied, the formation process and governing factors of the construction of SACs remain to be elucidated, making the regulation of metal‐metal and metal‐support interactions among single atoms difficult. Especially, two key challenges plagued the fabrication of high‐density SACs with a wide range of composition: how to incorporate high density of metal cations in MOFs substrate; and how to prevent the aggregation of these metal species into clusters and nanoparticles during the removal of ligands. As a result, only limited options of MOFs have been developed to deliver high‐density single atoms.[^11a^, ^14^] The elucidation of formation process of MOFs‐derived SACs is significant to guide the synthesis of next generation of high‐density SACs, which demonstrate high promise to create unique geometric and electronic structures.

Herein, we fully elucidated the formation process of high‐density single atoms with metal‐metal interactions by the combination of mutually supporting in situ/ex situ spectroscopic measurements and theoretical calculations. Firstly, we revealed that a high density of Pt cations can readily substitute Co cations in ZIF‐67 via a cation exchange process. This process conforms with Hard‐Soft Acid‐Base (HSAB) principle, which requires a stronger bond between guest metal and MOF ligand than the host metal‐ligand bond. In addition, we demonstrated that the Pt−Pt second‐shell coordination was gradually formed as a result of the evolution of first‐shell coordination during pyrolysis. Upon oxygen attack, the Pt−N bond was broken to remove the linking imidazole ligand, which is accompanied by the formation of a new Pt−O bond. This Pt‐ligand transition is preferentially achieved in comparison with that of host Co‐ligand, which is the driving force of forming Pt−O−Pt moiety and inducing Pt−Pt interactions between Pt single atoms. With both processes accomplished, high‐density Pt single atoms (≈6 weight %, ≈1.1 atom %) with Pt−Pt interactions were achieved within the lattice of cobalt oxide, which exhibited significantly enhanced catalytic activity. In contrast, hybrid systems against the two criterions generate isolated single atoms or segregated nanoparticles. This work provides fundamental insights for the construction of high‐density SACs with metal‐metal interactions.

## Results and Discussion

**Figure 1:**
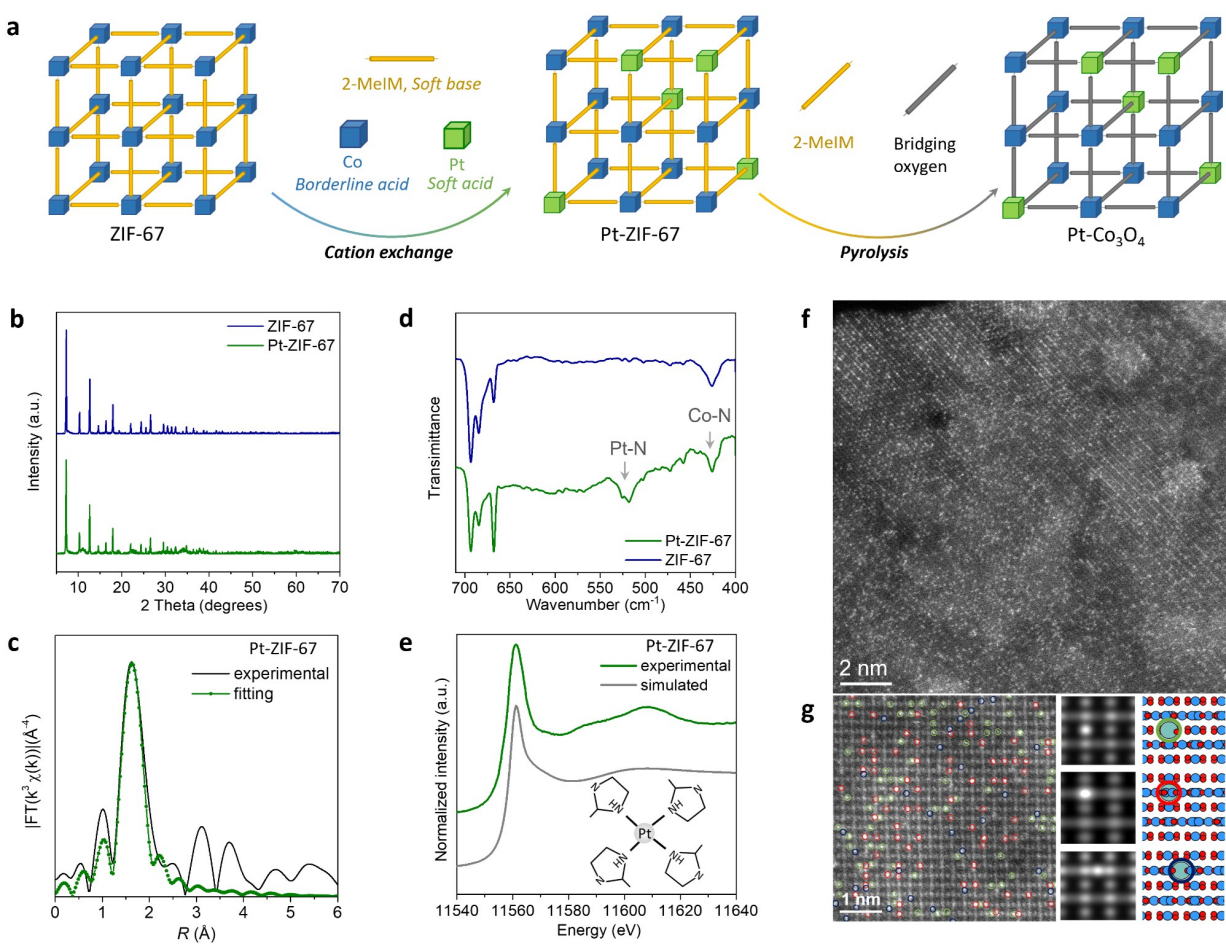
Cation exchange of Pt precursor with ZIF‐67. a) Schematic illustration of cation exchange of Co species in ZIF‐67 by Pt cations according to HSAB principle. b) XRD patterns of exchanged ZIF‐67 and pristine Pt‐ZIF‐67. c) Pt L_3_‐edge FT‐EXAFS magnitudes of the experimental and fitting spectra of Pt‐ZIF‐67. d) DRIFTs spectra of exchanged Pt‐ZIF‐67 and pristine ZIF‐67 with Co−N and Pt−N bands indicated by arrows. e) Comparison of experimental and simulated Pt L_3_‐edge XANES spectra of Pt‐ZIF‐67. The inset is the structure model adopted in the simulation. f) Atomic‐resolution HRSTEM image of Pt‐ ZIF‐67‐300 °C catalyst. g) Experimental HRSTEM image of Pt‐ ZIF‐67‐300 °C catalyst with Pt sites labelled by different colored circles and the corresponding simulated projected Z^2^‐maps of each type of substitutions along symmetry‐related <233> directions. Red, blue and cyan filled spheres represent oxygen, cobalt and platinum atoms, respectively.

Here we take the popular cobalt‐based zeolitic imidazolate frameworks‐67 (ZIF‐67) as a model platform, which consists of central Co^II^ ions and four coordinating 2‐methyl imidazole (2‐MeIm) ligands. A cation exchange process was performed on ZIF‐67 with platinum (Pt) precursor (potassium hexachloroplatinate hydrate, K_2_PtCl_6_⋅xH_2_O) in aqueous solution, the obtained hybrid is named as Pt‐ZIF‐67. As illustrated in Figure 1a, the cation exchange process is dominated by the strength of coordination bonds between metal cations and organic ligands, which act as Lewis acids and bases, respectively.^[15]^ According to the classic HSAB principle, the Lewis acids and bases are categorized into soft, hard and borderline species.^[16]^ It is widely accepted that soft bases prefer and bind well with soft acids while hard bases are more likely to strongly bind hard acids.^[17]^ The relatively labile interaction between the borderline divalent cobalt ions and soft imidazole ligands makes it possible for cation exchange by soft noble metal cations to form a stronger metal‐ligand coordination.

The X‐ray diffraction (XRD) patterns demonstrate nearly identical crystal structure of Pt‐ZIF‐67 and ZIF‐67 with no peaks related to Pt, indicating that Pt sites are incorporated in ZIF‐67 as single atoms and no nanoparticles were formed (Figure 1b). The Pt L_3_‐edge extended X‐ray absorption fine structure Fourier transform (FT‐EXAFS) and Wavelet transform (WT‐EXAFS) spectra were measured to characterize the local coordination structure of Pt sites. While FT‐EXAFS reveals one major peak at around R=1.6 Å, WT‐EXAFS shows a dominated local intensity maximum at about k=5.0 Å^−1^ which can be assigned to the first shell Pt−N coordination (Figure 1c and Figure S1). No signal of Pt−Pt coordination can be observed. The fitting result of FT‐EXAFS spectra indicates that the coordination number (CN) of Pt−N is 4.2, which is close to the CN of Co−N coordination (4.0) in pristine ZIF‐67, suggesting the successful substitution of Co cations by Pt species (Table S1). This is further supported by diffuse reflectance Fourier‐transform infrared spectroscopy (DRIFTs) measurements, which demonstrate that apart from Co−N band at 427 cm^−1^ in pristine ZIF‐67, an extra band at 520 cm^−1^ ascribing to Pt−N can be observed in Pt‐ZIF‐67 (Figure 1d).^[18]^ Accordingly, we proposed a local Pt‐ZIF‐67 structure model with Pt cation replaces Co cation and coordinates with four 2‐MeIm ligands. A good agreement between experimental and simulated Pt L_3_‐edge X‐ray absorption near edge structure (XANES) spectra verifies the rationality of the structural model (Figure 1e).

As a following step, we performed pyrolysis of Pt‐ZIF‐67 at 300 °C in air to remove the organic ligands and transform the host framework into cobalt spinel oxide (Co_3_O_4_). For convenience, the obtained hybrid is denoted as Pt‐ZIF‐67‐300 °C. The high‐angle annular dark‐field high‐resolution scanning transmission electron microscopy (HAADF‐STEM) imaging permits the atomic‐resolution structural elucidation of the hybrid (Figure 1f and Figure S2). High‐density of Pt single atoms with brighter contrast can be observed to integrate in the lattice of cobalt oxide host. In addition, several symmetry‐related projections of an identical doping configuration can be identified and labelled with different colors (Figure 1g, left). The well match between simulated Z^2^‐maps and experimental observations demonstrates that the Pt single atoms substitute octahedral Co (Co_oct_) sites within the Co_3_O_4_ lattice (Figure 1g, right).

**Figure 2:**
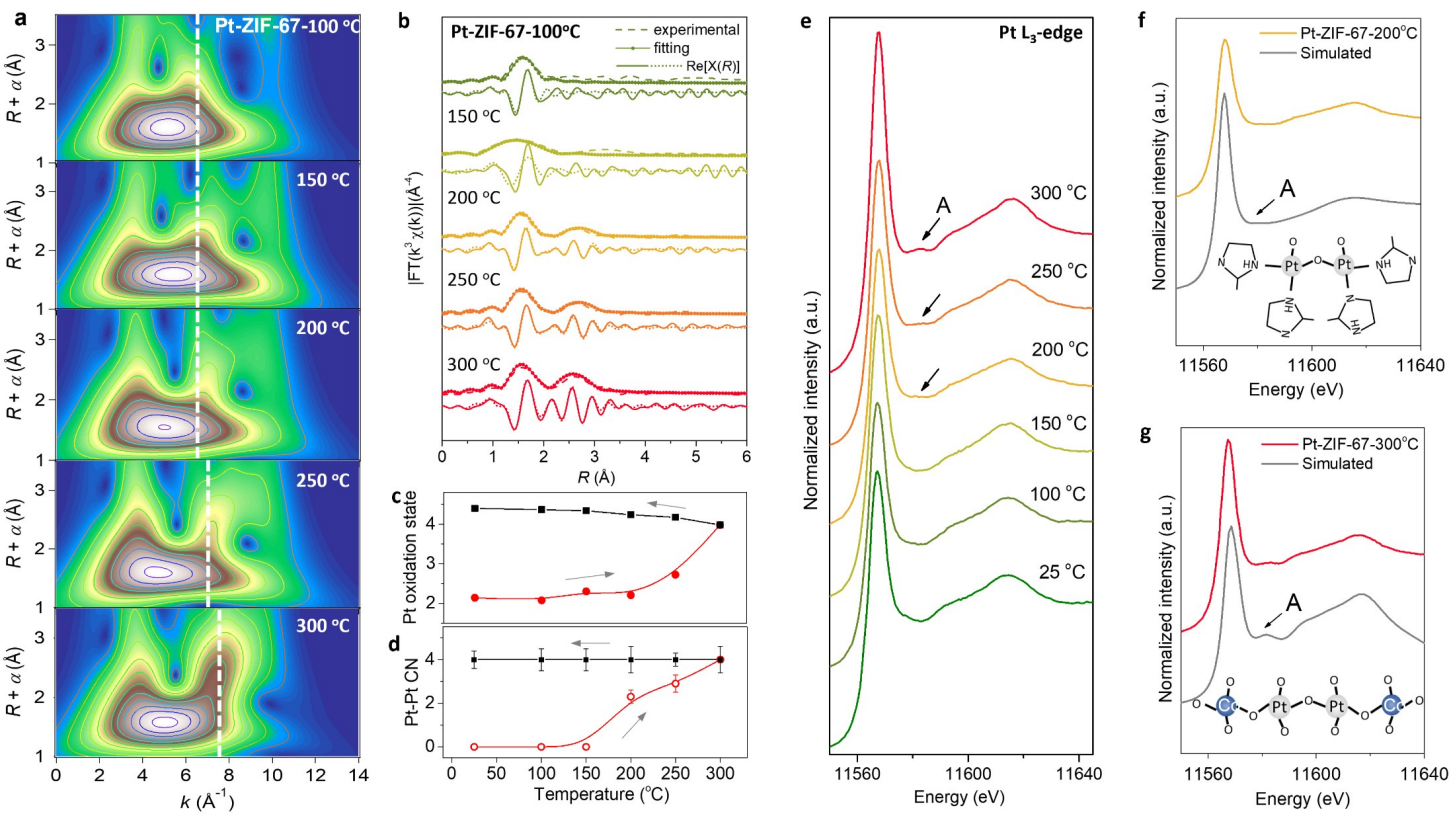
Evolution of Pt−Pt coordination during pyrolysis. a) In situ Pt L_3_‐edge WT‐EXAFS spectra of Pt‐ZIF‐67 pyrolyzed at different temperatures. The vertical dashed white lines highlight the gradual shift to higher k direction with increased temperature. b) In situ Pt L_3_‐edge FT‐EXAFS experimental spectra and the corresponding fittings of Pt‐ZIF‐67 under different pyrolysis temperatures. The solid and dotted lines represent the magnitudes and real parts of FT‐EXAFS, respectively, which is critical to validate the atom types selection for the different coordination shells. c) The changes of Pt oxidation states measured by white line areas from XANES spectra and d) CNs of Pt−Pt coordination shell fitted from FT‐EXAFS spectra on Pt‐ZIF‐67 under different temperatures during pyrolysis and following cooling process. e) In situ Pt‐L_3_ edge XANES spectra of Pt‐ZIF‐67 under different pyrolysis temperatures. The arrows highlight the feature peak A at ≈11 580 eV. Comparison of experimental and simulated Pt L_3_‐edge XANES spectra of f) Pt‐ZIF‐67‐200 °C and g) Pt‐ZIF‐67‐300 °C. The insets are the illustrative structure models adopted in the simulation.

To interpret the dynamic formation of high‐density Pt single atoms, we first focus on the evolution of Pt−Pt local coordination during the pyrolysis. In situ Pt L_3_‐edge WT‐EXAFS spectra were measured on Pt‐ZIF‐67 over the pyrolysis process. With the increase of temperature, the dashed lines in Figure 2a display that the second shell characteristic region exhibits a gradually increased scattering intensity as well as a shift to higher *k* direction. By comparing to the Pt foil and PtO_2_ references (Figure S1), this region can be ascribed to the Pt−Co/Pt second shell coordination. In addition, because the right‐shift of the region is strongly correlated with the increased scattering ability for the second shell atoms to photoelectron, it implies an initial formation of Pt−Co interactions and then emergence of Pt−Pt interactions among Pt single atoms, which is in good agreement with the HAADF‐STEM result (Figure S2).

Subsequently, in situ Pt L_3_‐edge FT‐EXAFS spectra were analyzed and fitted (Figure 2b, Figures. S3, S4). The first peak at 1.6 Å assigned to Pt−N/O first‐shell coordination demonstrates an interatomic distance of ≈2.0 Å and an increased CN from around 4.3 (before 250 °C) to 6.4 at 300 °C (Table S1). This is supported by a significantly increased white‐line area observed at 300 °C, corresponding to an increase of Pt oxidation state from ≈2.0 to ≈4.0 (Figure 2c and Figure S5). Another peak at 2.6 Å can be assigned to second shell coordination. The fitting results suggest the co‐existence of Pt−Co and Pt−Pt scatterings, both of which increased with the increase of pyrolysis temperature from around 200 °C to 300 °C. The gradually increased CN of the Pt−Pt coordination with an interatomic distance of ≈2.8 Å confirms the formation of Pt single atoms with Pt−Pt interactions (Figure 2d and Table S1). Additionally, the oxidation state and coordination environment of Pt sites was fully remained and kept unchanged during the following cooling process after pyrolysis (Figures 2c, 2d and Figures S6–S8).

Notably, in situ Pt‐L_3_ edge XANES spectra of Pt‐ZIF‐67 demonstrate that an additional feature peak A at ≈11 580 eV starts to emerge since 200 °C, which gets stronger in the following pyrolysis stage (Figure 2e). We inferred that the additional peak is related to the formation of paired Pt single atoms with Pt−Pt interactions. We proposed a few structure models, in which the linking 2‐MeIm between paired Pt single atoms were partially replaced by oxygen atoms. As is shown in Figure 2f and Figure S9, the calculated spectra are in a good agreement with the experimental spectra, suggesting the formation of Pt−O−Pt moiety during pyrolysis, which will be discussed in detail later. In addition, a well match can be achieved between Pt‐L_3_ XANES spectra at 300 °C and simulations on structure models with paired Pt singe atoms integrated in Co_3_O_4_ (Figure 2g and Figure S10). It is demonstrated that all the proposed structures are reasonable and can potentially exist solely or simultaneously in the corresponding sample, validating the formation of high‐density Pt single atoms with Pt−Pt interactions within the lattice of Co_3_O_4_. In addition, XANES spectra of Pt clusters with direct Pt−Pt bonding were theoretically simulated, which is in marked contrast the experimental spectra, excluding the possibility of forming Pt clusters (Figure S11). Therefore, we proved that the cation exchange‐pyrolysis strategy can be applied to prepare Pt single atoms with Pt−Pt interactions via a Pt−O−Pt moiety.

**Figure 3:**
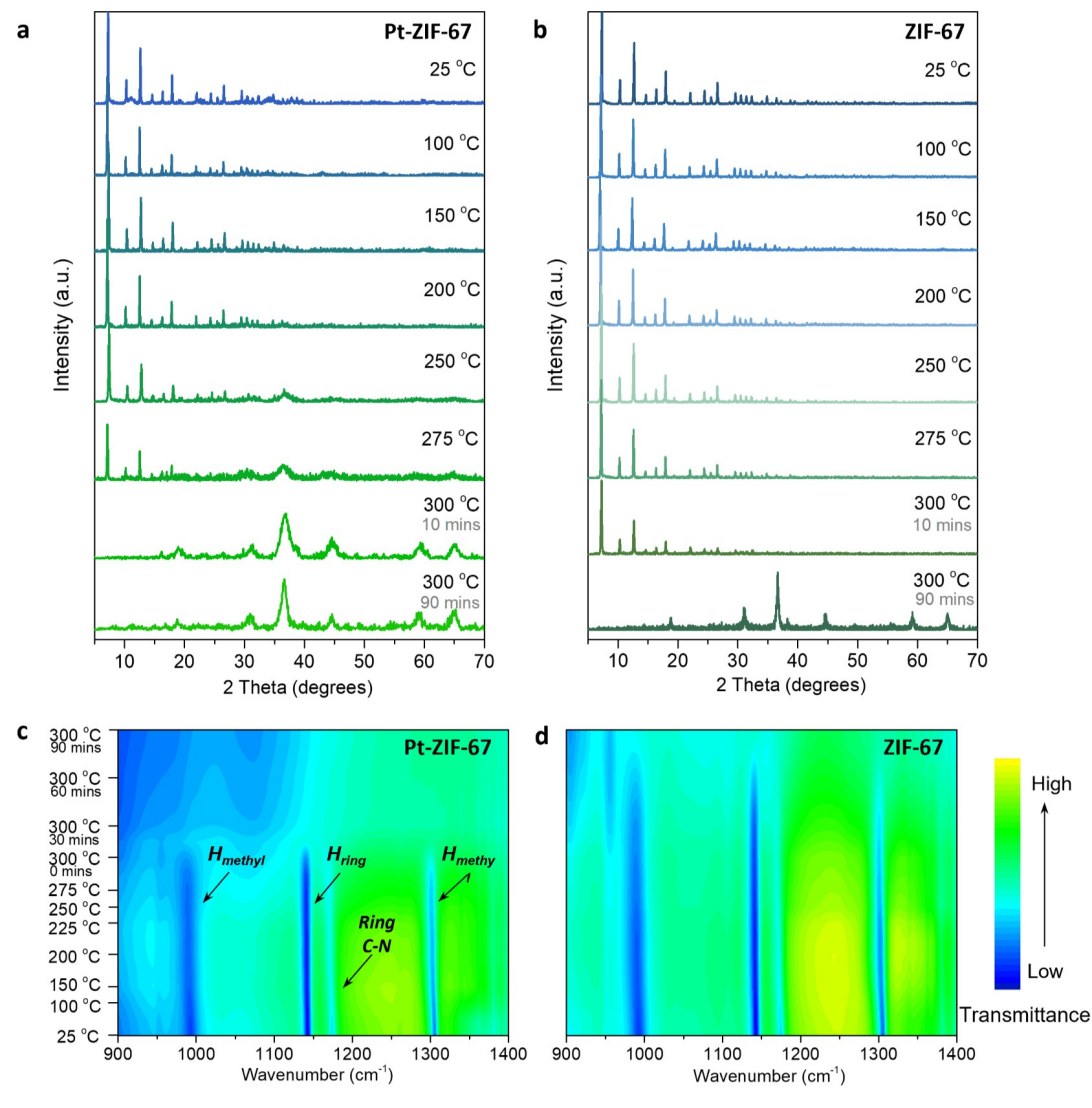
Evolution of organic ligand during pyrolysis. Ex situ XRD patterns on a) Pt‐ZIF‐67 and b) ZIF‐67 catalysts pyrolyzed under different temperatures, the grey labels of 10 mins and 90 mins indicate the time held at 300 °C. In situ temperature‐dependent DRIFTs spectra of c) Pt‐ZIF‐67 and d) pristine ZIF‐67 with characteristic peaks indicated by arrows and labels.

Then we investigated the evolution of ligands during pyrolysis. Ex situ XRD patterns of exchanged Pt‐ZIF‐67 show that the framework was well retained with the increase of temperature from 25 °C to 200 °C (Figure 3a). An additional new phase corresponding to cobalt spinel oxide (Co_3_O_4_, *Fd3m* symmetry) was observed after 250 °C until the ZIF‐67 phase completely disappeared at 300 °C. In contrast, the pristine ZIF‐67 exhibited much higher thermostability with a phase transformation to Co_3_O_4_ takes place after held at 300 °C for around 90 mins (Figure 3b).

In situ temperature‐dependent DRIFTs measurements were performed to investigate the evolution of organic ligand. As is shown in the contour maps in Figures 3c, 3d and spectra in Figures S12–S13, a few characteristic peaks of ZIF‐67 framework can be observed in both Pt‐ZIF‐67 and ZIF‐67.^[19]^ With the increase of pyrolysis temperature, these peaks in Pt‐ZIF‐67 remain almost unchanged before a sudden disappearance at around 300 °C. This suggests that even though the host frameworks have collapsed since 250 °C, the organic ligands were retained until the fully structure decomposition and transition to Co_3_O_4_ at 300 °C. In contrast, ZIF‐67 did not undergo such structure decomposition until held at 300 °C for more than 60 mins. The XRD and DRIFTs observations confirm that the fully phase transition of Pt‐ZIF‐67 occurs earlier than that of pristine ZIF‐67, both of which were accompanied by decomposition of 2‐MeIm ligand. This result suggests that the incorporated Pt sites are possibly more vulnerable to be attacked by oxygen and therefore facilitate the phase transition to Co_3_O_4_.

**Figure 4:**
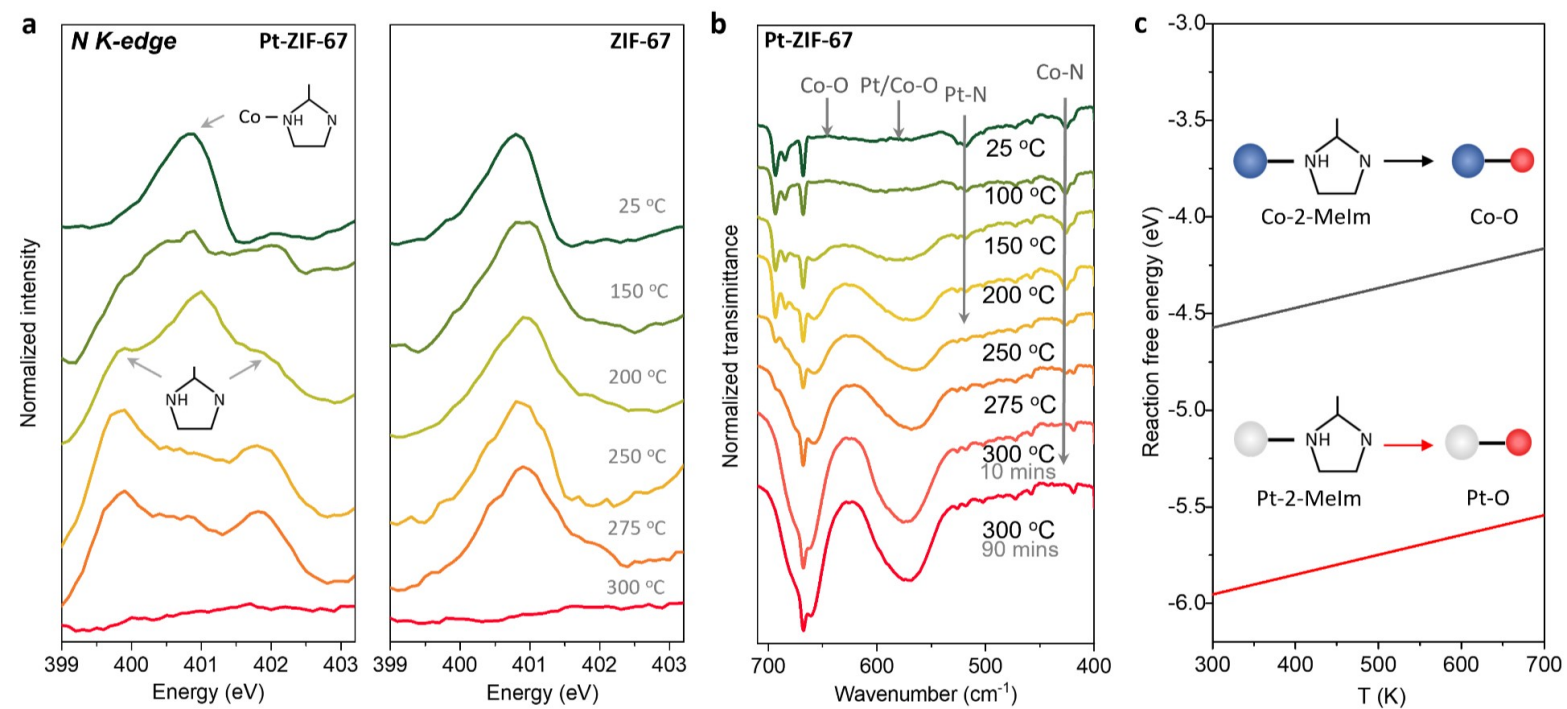
Evolution of metal‐ligand coordination during pyrolysis. a) Ex situ N‐K edge XANES spectra on Pt‐ZIF‐67 (left) and pristine ZIF‐67 (right). The main species of Co‐2‐MeIm and pristine 2‐MeIm are indicated with arrows and labels. b) Ex situ DRIFTs spectra in low wavenumber region of Pt‐ZIF‐67 under different pyrolysis temperatures. The Pt−N/Co−N and Pt−O/Co−O bands are indicated by grey arrows. c) Comparison of reaction free energies on Pt‐ligand to Pt−O transition and Co‐ligand to Co−O transition, which is obtained by DFT calculations and extended to whole temperature range. The insets are schematic illustrations of the transitions.

To fully elucidate driving force of the above construction of Pt−Pt interaction and cleavage of organic ligand, the local metal‐ligand coordination evolution of Pt sites was interpreted. Firstly, a local investigation of the M−N coordination was achieved by ex situ N‐K edge soft XANES measurements, which reveal the change of nitrogen coordination in 2‐MeIm ligand during pyrolysis. In contrast to pristine ZIF‐67 with nearly unchanged Co−N peak (400.9 eV), the Pt‐ZIF‐67 exhibit decreased intensity of Co−N species together with the emergence of two peaks (399.8 eV, 402.0 eV) corresponding to nitrogen species in 2‐MeIm (Figure 4a).^[20]^ Such transition from Co‐2‐MeIm to pristine 2‐MeIm species demonstrates the cleavage of Co−N bond in Pt‐ZIF‐67 during the pyrolysis. In addition, ex situ O‐K edge XANES spectra suggests that the change of N species was accompanied by the formation of Co−O bond, which occurs at a much lower temperature in Pt‐ZIF‐67 than in ZIF‐67 (Figure S14). Beside ligand, we then investigated the evolution of metal‐related peaks at low wavenumber range of DRIFTs spectra (Figure 4b). It is demonstrated that the Pt−N band in Pt‐ZIF‐67 shows rapidly reduced intensity with the increase of temperature until completely disappeared before 200 °C, while a similar process happens on Co−N band at a higher temperature up to 275 °C. Simultaneously, the emergence of Pt−O and Co−O bands can be observed at around 570 cm^−1^ and 660 cm^−1^.^[21]^ In contrast, the pristine ZIF‐67 exhibits postponed transition from Co−N to Co−O at 300 °C (Figure S15). These observations agree well with the XANES spectra, confirming the preferential cleavage of Pt−N bond in comparison with Co−N bond. To reveal the origin of preferred Pt‐ligand transition to Pt−O in comparison with that of Co‐ligand bonds, we conducted density function theory (DFT) calculations to measure the free energy change on reactions from metal‐ligand to metal‐oxygen (see details in Methods section). It is indicated that the Pt‐2‐MeIm transition to Pt−O exhibits a more negative reaction free energy than that of Co‐2‐MeIm to Co−O over the entire temperature range (Figure 4c and Figure S16). This suggests the more favorable thermodynamics of Pt‐ligand to Pt−O transition, explaining why Pt‐ligand is more vulnerable to oxygen attack during pyrolysis. This permits the formation of Pt−Pt interactions between Pt single sites, which can be immobilized as Pt−O−Pt moiety within the lattice of Co_3_O_4_ during the subsequent oxidation of host framework. Therefore, it is proved that the formation of high‐density Pt single atoms with Pt−Pt interactions is mediated by a Pt−O−Pt moiety during pyrolysis. The combination of XRD, DRIFTs and XANES investigations during pyrolysis elucidates the ligand evolution of Pt‐ZIF‐67, which undergoes cleavage of M−N bond and formation of M−O coordination. It is also concluded that the free energy difference between guest metal‐ligand transition and host metal‐ligand transition is a critical factor in the formation of paired metal single atoms.

**Figure 5:**
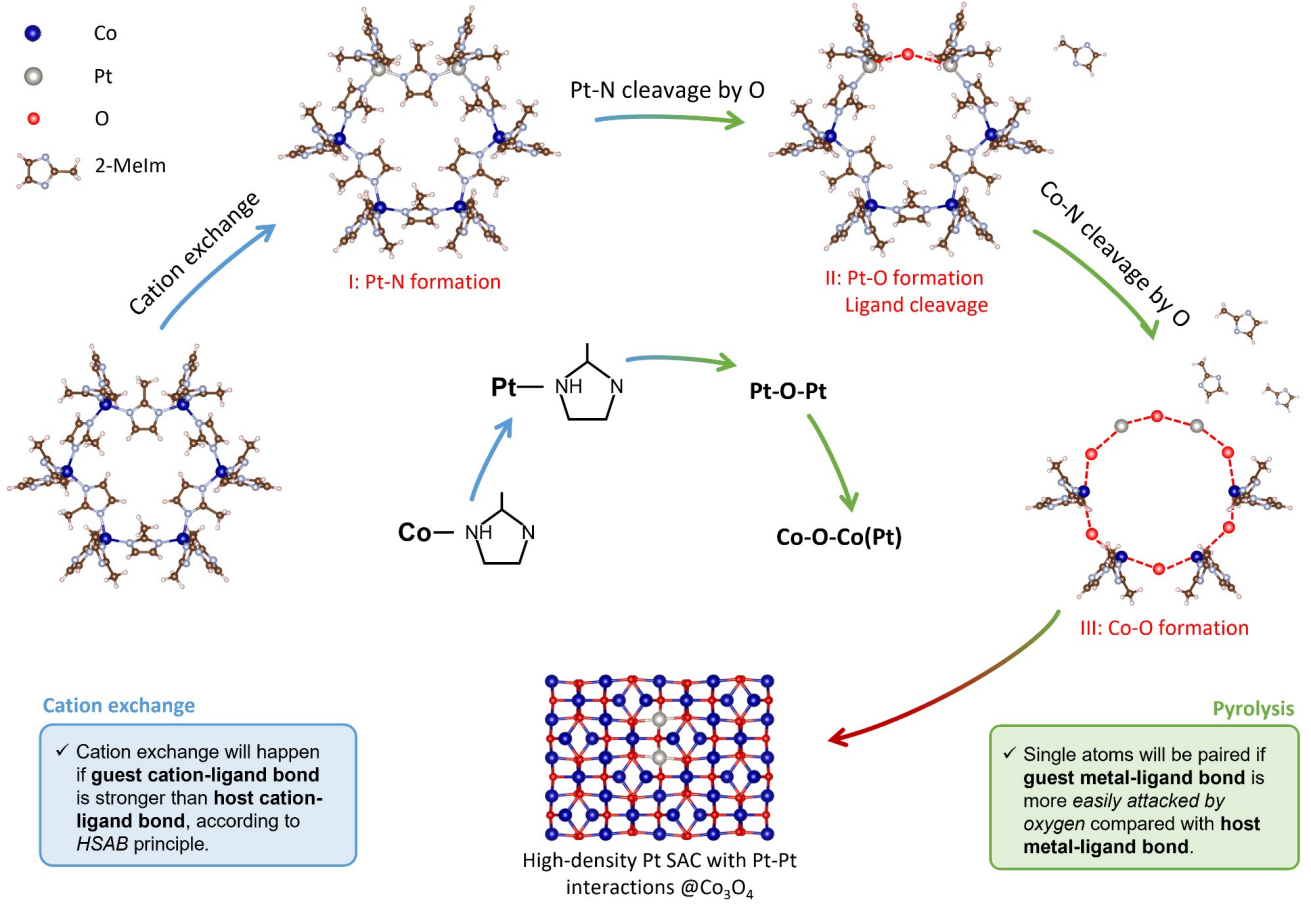
Formation mechanism of high‐density Pt single atoms with Pt−Pt interactions. Outer circle is the schematic illustration of formation process of high‐density Pt single atoms with Pt−Pt interactions in Co3O4. Firstly, Co cations were exchanged by Pt cations to form Pt−N4 coordination in ZIF‐67 (Step I). Then, during pyrolysis process, Pt−N bond was preferably attacked by oxygen to form Pt−O−Pt moiety (Step II) and then Co−N bond was attacked by oxygen to produce cobalt oxide (Step III). Inner circle is the corresponding molecule structure evolution during the synthesis procedure. The criterions of cation exchange and pyrolysis processes are summarized in blue and green boxes on the bottom, respectively.

On the basis of in situ/ex situ spectroscopic measurements, XANES simulation and DFT calculations, we have achieved a detailed picture of formation processes of high‐density Pt single atoms, including cation‐exchange and thermally induced evolution of Pt−Pt interactions, organic ligands cleavage, and Pt‐ligand coordination. The formation mechanism and two criterions can be therefore clarified (Figure 5). Firstly, the successful proceeding of cation exchange process results in the substitution of Co cations in ZIF‐67 by Pt cations, which coordinate with four 2‐MeIm ligands via Pt−N_4_ coordination (Step I: Pt−N bond formation). An important consideration in this process is HSAB principle, which determines the possibility and degree of cation exchange reaction. The cation exchange will take place if guest metal can form a stronger metal cation‐ligand bond (e.g., soft metal‐soft ligand bond, hard metal‐hard ligand bond) compared with the host metal cation‐ligand bond, resulting in a high content of guest metal cations incorporated in the host MOFs. Too low content of guest metal cations would easily lead to isolated single atoms.

During the subsequential pyrolysis process, the Pt‐ligand bond is preferably attacked by oxygen molecules to form Pt−O bond with ligand cleavage (Step II). This makes it possible for the Pt single atoms to exhibit Pt−Pt interaction via a Pt−O−Pt moiety. The later Co‐ligand transition to Co−O results in oxidation of cobalt species and the immobilization of paired Pt single atoms in the lattice of Co_3_O_4_ (Step III). Therefore, it is proposed that if the guest metal‐ligand bond is attacked by oxygen before it happens on host metal‐ligand bond, the guest metal species can be coordinated by bridge oxygen sites to form paired single atoms with metal‐metal interactions. Otherwise, if the guest metal‐ligand bond is not readily attacked by oxygen, the guest metal species are inclined to aggregate into nanoparticles at a higher temperature when the collapse and decomposition of host framework happens. With both criterions satisfied, high‐density Pt sites can be incorporated in ZIF‐67 via cation exchange with Pt−Pt interactions within the lattice of cobalt oxide via pyrolysis.

**Figure 6:**
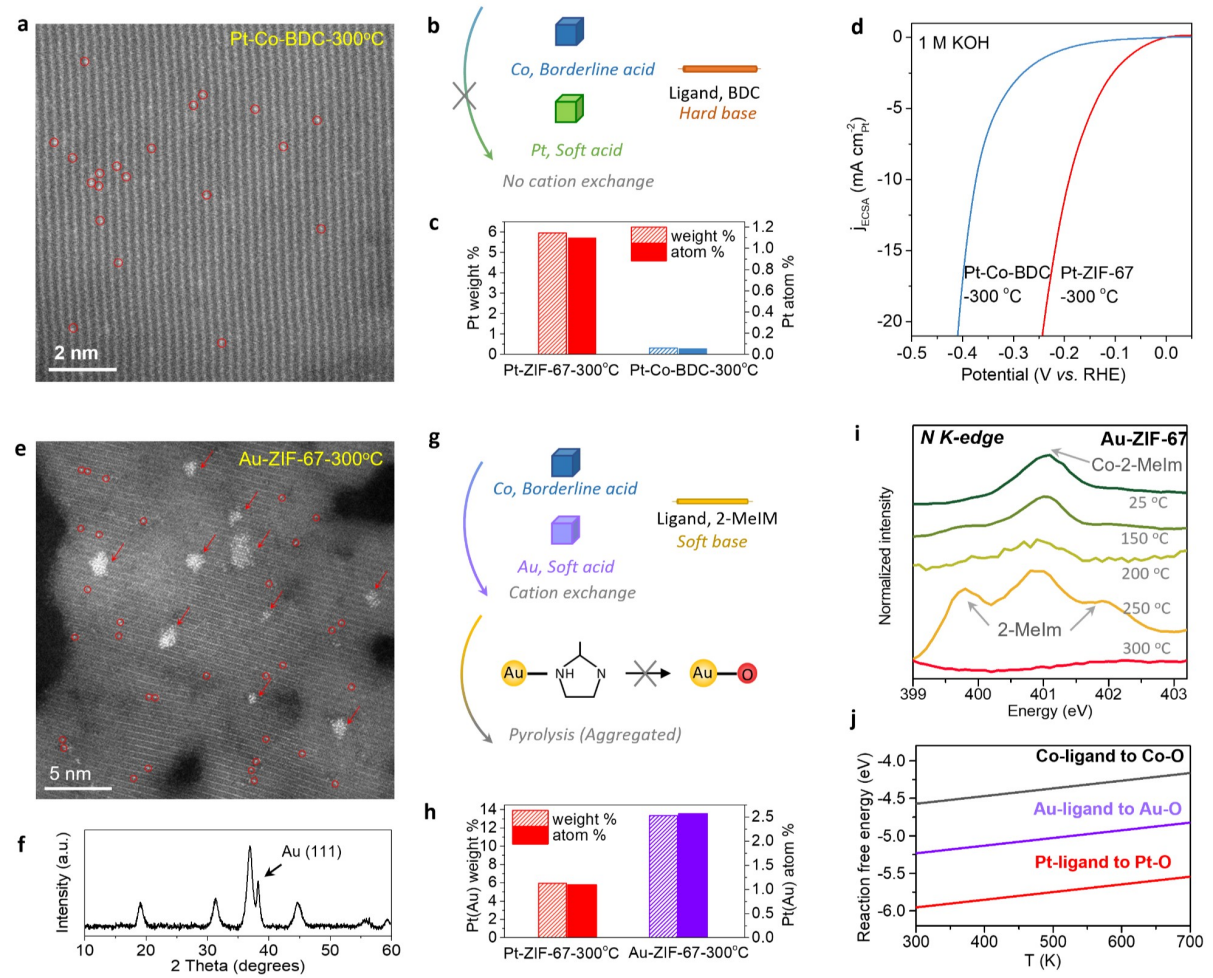
Validations of proposed formation mechanism. a) HAADF‐STEM image of Pt‐Co‐BDC‐300 °C catalyst with low content of Pt single atoms indicated by red circles. b) Schematic illustration of cation exchange of Co species in Co‐BDC by Pt cations according to HSAB principle. c) Pt weight % (left y‐axis) and Pt atom % (right y‐axis) of Pt‐ZIF‐67‐300 °C and Pt‐Co‐BDC‐300 °C calculated by ICP‐MS measurements. d) Polarization curves of HER electrocatalysis normalized to ECSAs in Ar‐saturated 1 M KOH media for Pt‐ZIF‐67‐300 °C and Pt‐Co‐BDC‐300 °C catalysts. e) HAADF‐STEM image of Au‐ZIF‐67‐300 °C catalyst with Au nanoparticles indicated by red arrows and Au single atoms indicated by red circles. f) XRD pattern of Au‐ZIF‐67‐300 °C, which shows a peak corresponding to Au (111) (PDF#: 65‐2870) in addition to those of Co_3_O_4_. g) Schematic illustration of cation exchange and pyrolysis processes of Au‐ZIF‐67, the latter process leads to aggregation of Au sites. h) Pt (Au) weight % (left y‐axis) and Pt (Au) atom % (right y‐axis) of Pt‐ZIF‐67‐300 °C and Au‐ZIF‐67‐300 °C calculated by ICP‐MS measurements. i) Ex situ N‐K edge XANES spectra on Au‐ZIF‐67 under different pyrolysis temperatures, which indicate that the Co‐2‐MeIm peak in Au‐ZIF‐67 was well retained over pyrolysis process until the co‐existence of 2‐MeIm bonds were observed at 250 °C. j) Comparison of reaction free energies on Au‐ligand to Au−O transition with those of Co and Pt counterparts, which is obtained by DFT calculations and extended to whole temperature range.

To validate the proposed formation mechanism, two control samples were designed specific to the two criterions. Firstly, a Co‐based MOF with hard base ligand (terephthalic acid, 1,4‐H_2_BDC) was adopted as the host. The obtained Pt‐Co‐BDC‐300 °C hybrid shows very low content of fully isolated Pt single atoms within the lattice of Co_3_O_4_ (Figure 6a, Figures S17, S18). According to HSAB principle, the soft Pt cation exhibit an even weaker metal‐ligand bond with the hard BDC ligand in comparison with that of Co, leading to an unlikely Pt cation exchange in Co‐BDC MOF (Figure 6b). Inductively coupled plasma mass spectrometry (ICP‐MS) measurements demonstrate only ≈0.31 weight % Pt in Pt‐Co‐BDC hybrid either after cation exchange or pyrolysis, in stark contrast to that of Pt‐ZIF‐67 hybrid (≈5.95 weight %) (Figure 6c and Table S2). Further, taking hydrogen evolution reaction (HER) electrocatalysis in alkaline media as a model reaction, we investigated the effect of Pt−Pt interactions between Pt single atoms. Comparison with Pt‐Co‐BDC‐300 °C with isolated Pt single atoms, the Pt‐ZIF‐67‐300 °C with high‐density Pt single atoms exhibit a much higher HER apparent activity as well as specific activity normalized to electrochemical active surface areas (ECSAs) of Pt sites (Figure 6d, S19, S20). This result suggests that the formation of metal‐metal interactions among single atoms may provide unique adsorption configuration and electronic structure for reaction intermediates, which is beneficial for improving catalytic activity of SACs.

On the other hand, another type of soft noble metal cation, Au, was adopted as guest cations to ZIF‐67. The HAADF‐STEM image of the obtained Au‐ZIF‐67‐300 °C hybrid shows that most of the Au species exist as Au nanoparticles while only few was remained as Au single sites (Figure 6e and Figure S21). This is also confirmed by XRD pattern that shows an evident peak of Au (111) in addition to the characteristic peaks corresponding to Co_3_O_4_ (Figure 6f). As illustrated in Figure 6g, a successful cation exchange between Au and ZIF‐67 is expected and validated by ICP‐MS. However, the Au‐ZIF‐67‐300 °C exhibits aggregation of high‐content Au (≈13.32 weight %) during the following pyrolysis process (Figure 6h). Ex situ XANES spectra show that Au‐ligand is not readily attacked by oxygen during pyrolysis until around 250 °C (Figure 6i and Figure S22). This is explained by DFT calculations, which demonstrate that the free energy change of the Au‐ligand transition to Au‐O process is much closer to that of Co species in comparison with that of Pt (Figure 6j and Figure S23). This leads to difficulty in forming paired Au‐O‐Au moiety and tendency of Au sites to aggregate into Au nanoparticles.

## Conclusion

In conclusion, we unambiguously elucidated the construction of SACs via MOF pyrolysis by focusing on the chemical bond cleavage and formation. The combination of diverse in situ and ex situ spectroscopic measurements critically revealed the evolution of cation exchange, Pt−Pt interaction, organic ligands cleavage, and Pt‐ligand coordination formation steps during the construction of high‐density Pt single atoms. We clarified the determining factors in the two stages of the synthesis process. On one hand, the incorporation content of guest metal cations in host MOF via cation exchange is governed by the cation‐ligand interaction according to HSAB principle. On the other hand, the dynamic regulation of guest metal‐ligand transition to guest metal‐oxygen during pyrolysis process promotes the formation of metal‐oxygen‐metal moiety, which is a key component to induce metal‐metal interactions between high‐density metal single atoms. This work provides fundamental understandings of the general construction of noble metal and transition metal SACs. It is expected to direct the rational synthesis of high‐density single atoms with flexible geometric structure, inter‐site synergistic effects and remarkable catalytic performance.

## Conflict of interest

The authors declare no conflict of interest.

1

## Supporting information

As a service to our authors and readers, this journal provides supporting information supplied by the authors. Such materials are peer reviewed and may be re‐organized for online delivery, but are not copy‐edited or typeset. Technical support issues arising from supporting information (other than missing files) should be addressed to the authors.

Supporting Information

## Data Availability

The data that support the findings of this study are available from the corresponding author upon reasonable request.
