# Supplementary material for: The Dynamic Formation from Metal‐Organic Frameworks of High‐Density Platinum Single‐Atom Catalysts with Metal‐Metal Interactions
Source: Angew Chem Int Ed Engl. 2022 Oct 27;61(48):e202213412. doi: 10.1002/anie.202213412 (PMC9828475; doi:10.1002/anie.202213412)
Supplement: Supplementary file 1 — Supporting Information [file ANIE-61-0-s001.pdf]

Angewandte Chemie
Eine Zeitschrift der Gesellschaft Deutscher Chemiker

Supporting Information

The Dynamic Formation from Metal-Organic Frameworks of High-Density Platinum Single-Atom Catalysts with Metal-Metal Interactions

J. Shan, J. Liao, C. Ye, J. Dong, Y. Zheng*, S.-Z. Qiao**

Material Synthesis. ZIF-67 nanocrystals were synthesized by a surfactant-mediated method reported previously.[1] In a typical procedure, 580 mg of cobalt (II) nitrate hexahydrate ($Co(NO_3)_2{\cdot}6H_2O$) was dissolved in 20 mL of deionized (DI) water containing 30 mg of hexadecyltrimethylammonium bromide (CTAB). Then, this solution was injected into 140 mL of aqueous solution with 9.08 g of 2-methylimidazole and stirred at room temperature for 60 min. The purple precipitate was collected by centrifugation and washed with ethanol five times.

Co-BDC was synthesized according to previous literature.[2] In a typical procedure, 124.6 mg of Benzenedicarboxylic acid (BDC) was dissolved into a mixture of 32 mL of *N, N*-dimethylformamide (DMF), 2 mL of ethanol and 2 mL of DI water. Next, 218.3 mg of $Co(NO_3)_2{\cdot}6H_2O$ was dissolved in the solution. Then, 0.8 mL Triethylamine (TEA) was quickly added in the mixture, a uniform colloidal suspension can be obtained after stirring for 5 mins. The colloidal solution was ultrasonicated overnight before centrifuged and washed with ethanol for 5 times.

In a cation exchange process, 100 mg of ZIF-67 nanocrystals were dispersed in 50 mL of DI water, then 9.5 mg (2 mmol L^{-1}) of potassium hexachloroplatinate (IV) hydrate ($K_2PtCl_6{\cdot}xH_2O$) were dissolved in 10 mL of DI water and added into the ZIF-67 solution slowly under stirring conditions. The reaction was quenched by centrifuging the suspension after 3 hours, the Pt-ZIF-67 precipitate was collected and washed twice with DI water and three times with ethanol. The Pt-ZIF-67-300 °C nanocrystals were obtained after drying the precipitate at 60 °C overnight in a vacuum oven and pyrolyzed at 300 °C in air for 4 hours. Au-ZIF-67 was achieved by a similar procedure with applying 2 mmol L^{-1} of gold (III) chloride hydrate ($HAuCl_4{\cdot}xH_2O$) aqueous solution in cation exchange process. Pt-Co-BDC-300 °C was prepared by similar cation exchange process with 10 mL of $K_2PtCl_6{\cdot}xH_2O$ (2 mmol L^{-1}) aqueous solution and 50 mL of Co-BDC solution (2 mg mL^{-1}), followed by centrifuged and washed with ethanol and pyrolyzed in air at 300 °C for 4 hours.

Materials characterization. XRD data was collected on a Rigaku MiniFlex 600 X-Ray Diffractometer. The soft XAS measurements were performed at the SXR beamline in Australian Synchrotron Radiation Facility, Melbourne. Ex-situ characterizations were conducted on samples pyrolyzed at different temperatures from 100 to 300 °C. The holding time was 15 mins at each temperature in the range of 100-275 °C and 10/60/90 mins for 300 °C. ICP-MS analysis was conducted using an Agilent 7500cx instrument with attached laser ablation system. HAADF-STEM images were recorded by using a FEI Titan G2 80-300

microscope at 300 kV equipped with a probe corrector. Projected Z^2-map simulations were performed by using the qSTEM program.[3]

DRIFTs measurements. DRIFTs measurements were performed using a Nicolet iS-50 (Thermo Scientific) with an MCT-B detector. 2 mg of the catalyst was mixed and finely ground with 98 mg of KBr powder. The mixed sample was vacuum dried 70 °C overnight to remove water and impurities. The mixture was placed in Praying Mantis DRIFT accessory (Harrick Scientific) and purged by compressed air during pyrolysis. All DRIFTs measurements were acquired by averaging 64 scans at a spectral resolution of 4 cm^{-1}. In-situ DRIFTs measurements were conducted at a heating rate of 2 °C/min and held at 100, 150, 200, 250 °C for 15 mins and 300 °C for 90 mins.

In-situ XAS measurements. The Pt L_3-edge XAS measurements were performed at the beamline of 4B9A beamline in Beijing Synchrotron Radiation Facility. The X-ray was monochromatized by a double-crystal Si (111) monochromator. The incident and transmitted X-ray intensities were monitored by using standard ion chambers, and the monochromator was detuned to reject higher harmonics. In-situ XAS measurements were conducted with a home-made furnace at a heating rate of 2 °C/min and held at 100, 150, 200, 250 °C for 15 mins and 300 °C for 90 mins. While the XAS raw data were background subtracted, normalized, and Fourier transformed by standard procedures within the ATHENA program, the least-squares curve fitting analysis of the EXAFS data was carried out using the ARTEMIS program.[4] The Pt L_3-edge theoretical XANES calculations were carried out with the FDMNES code in the framework of real-space full multiple-scattering scheme. Muffin-tin approximation for the potential was used.[5] Satisfactory convergence for the cluster size had been achieved.

DFT calculations. DFT calculations were carried out using the Vienna ab-initio Simulation Package (VASP).[6] The exchange-correlation interaction was described by generalized gradient approximation (GGA) with the Perdew-Burke-Ernzerhof (PBE) functional.[7] The projector augmented wave (PAW) pseudopotential scheme is used and the force and energy convergence tolerance values with respect to plane-wave cutoff and K-point density were set to be 0.01 eV $Å^{-1}$ and 10^{-5} eV, respectively.[8] Free energies and formation energies of all the compounds were obtained using the GGA-DFT plus Hubbard-U framework (GGA+U). Ferromagnetic spin-polarized calculations were employed for all magnetic materials. The GGA+U calculations were performed using the model proposed based on the TEM images

with the U_{eff} (U_{eff} = Coulomb U - exchange J) values of 4.4, 6.7 and 3.2 eV for Co^{2+}, Co^{3+} and Pt, respectively.[9]

Co_2(2-MeIm)$_7$ + 1/2 O_2 → Co_2(2-MeIm)$_4$O + 3 (2-MeIm)

The reaction free energy of this reaction can be represented by ΔG reaction = $G_{2\text{-MeIm}}$ + $G_{Co2(2\text{-MeIm})4O}$ − 1/2 G_{O2} − $G_{Co2(2\text{-MeIm})7}$. Among the reactants and products, the Co_2(2-MeIm)$_7$, the Co_2(2-MeIm)$_4$O and the 2-MeIm can be treated as solid state. Here, we assign the enthalpy and entropy as H = E^{DFT} and S = 0 (at moderate temperatures).[10] Thus, the Gibbs free energy ($G_{i(s)}$) of a solid element i can be represented by DFT calculations as $G_{i(s)} = E^{DFT}$ i(s). The $G_{O2(g)}$ at the standard state can be presented as $2\mu_{O(g)} = G_{O2(g)}$ = EDFT O_2(g) + EZPE O_2(g) + RT - TSexp O_2(g) ,where Sexp O_2(g) = 205.152 J mol^{-1} K^{-1} is taken from the literature.[11] Here, EDFT O_2(g) = -8.746 eV and EZPE O_2(g) = 0.105 eV can be obtained by DFT calculations.

Electrochemical measurements. Electrocatalyst ink was prepared by dispersing a freshly synthesized catalyst powder (2 mg) in a solution containing distilled water (Milli-Q, 965 μL) and 5 weight % Nafion solution (35 μL) followed by ultrasonication for 2 hr. 10 μL of catalyst ink was then deposited onto a polished Au-electrode (diameter = 5 mm, area = 0.196 cm^2, Pine Research Instrument). All electrochemical experiments were carried out in a three-electrode glass cell with an Au-wire as the counter electrode and an Ag/AgCl as the reference electrode (Pine Research Instrument). The reference electrode was calibrated in Ar-saturated 1 M KOH electrolyte. All potentials were converted to the reversible hydrogen electrode (RHE) and corrected for iR-compensation. The HER measurements were conducted in Ar-saturated 1 M KOH electrolyte with a CHI potentiostat (CHI 760E) at a rotating speed of 1600 rpm. The polarization curves of the catalysts were obtained with scan rates of 5 mV s^{-1}. The ECSAs of the catalysts were determined by CO stripping voltammograms.[12] The electrolyte was bubbled with 20 % CO in argon for 30 min with the working electrode held at 0.2 V *vs.* RHE, followed by argon purging for 20 min to remove the excess CO. Then the CO stripping voltammograms were collected in the potential range from around 0 V to 1.2 V *vs.* RHE during Ar bubbling. The ECSAs values were calculated based on the following equation[13]:

$$\text{ECSA} = \frac{Q_{CO\_strip}}{Q_{CO\_strip}{}^{theo}} = \frac{Q_{CO\_strip}}{420\ \mu\text{C cm}^{-2}}$$

where Q_{CO_strip} is the integrated charge obtained from CO-stripping curves, the $Q_{CO\_strip}^{theo}$ is the theoretical value for a two-electron transfer assuming the oxidation of one CO to CO_2 per Pt atom.

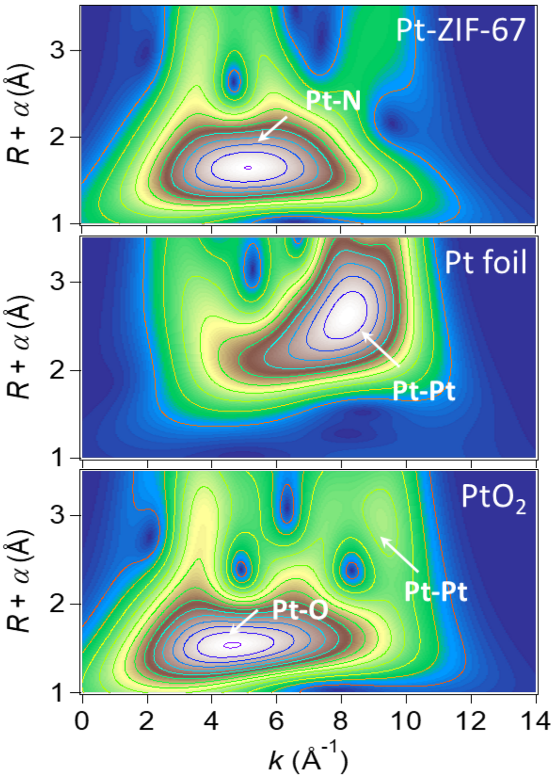

Fig. S1. Pt L_3-edge WT-EXAFS spectra of Pt-ZIF-67, Pt foil and PtO_2 references. It can be observed that while the intensity maximum near 5.0 $Å^{-1}$ can be assigned to the Pt-O scattering, the intensity maxima near 8.0 $Å^{-1}$ can be associated with the Pt-Pt contributions. Thus, the maximum at approx. R = 1.6 Å and k = 5.1 $Å^{-1}$ in Pt-ZIF-67 corresponds to Pt-N scattering.

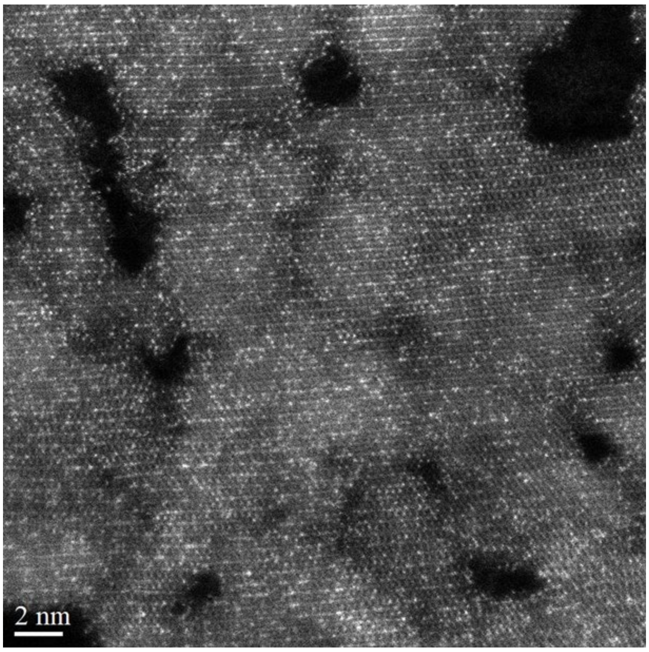

Fig. S2. HAADF-STEM image of Pt-ZIF-67-300 °C.

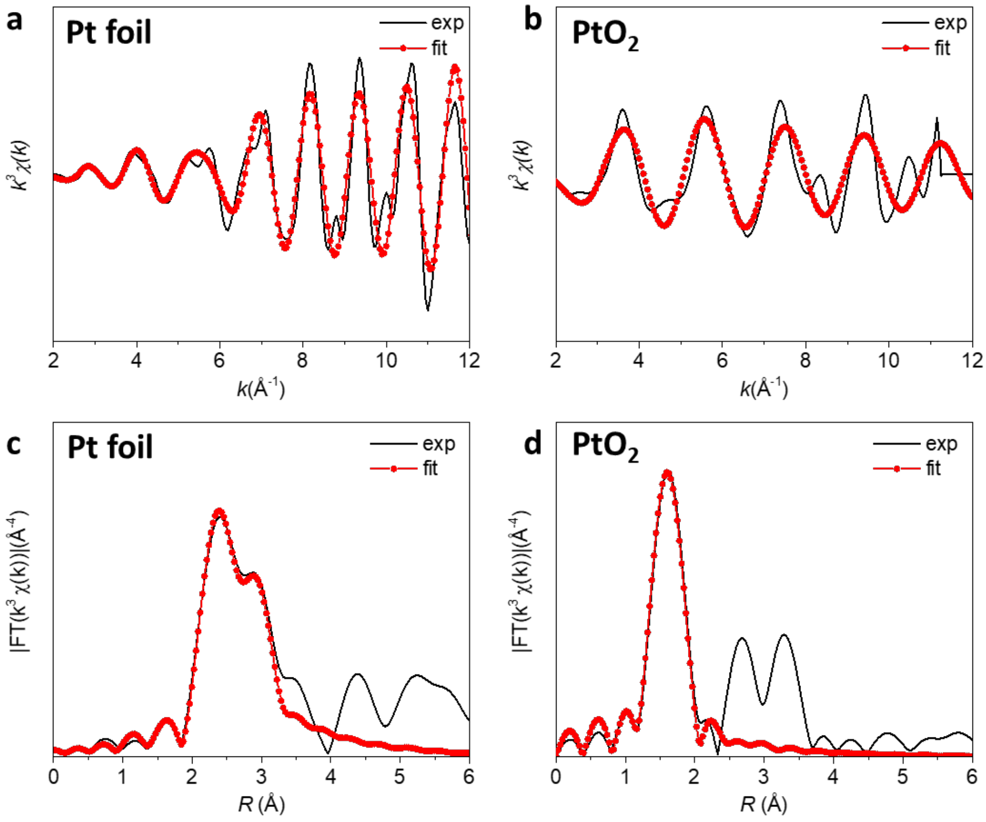

Fig. S3. (a, b) Pt L_3-edge experimental (exp) and fitting (fit) EXAFS spectra of Pt foil and PtO_2 references. (c, d) Fourier-transformed magnitudes of Pt L_3-edge experimental (exp) and fitting (fit) EXAFS spectra of Pt foil and PtO_2 references.

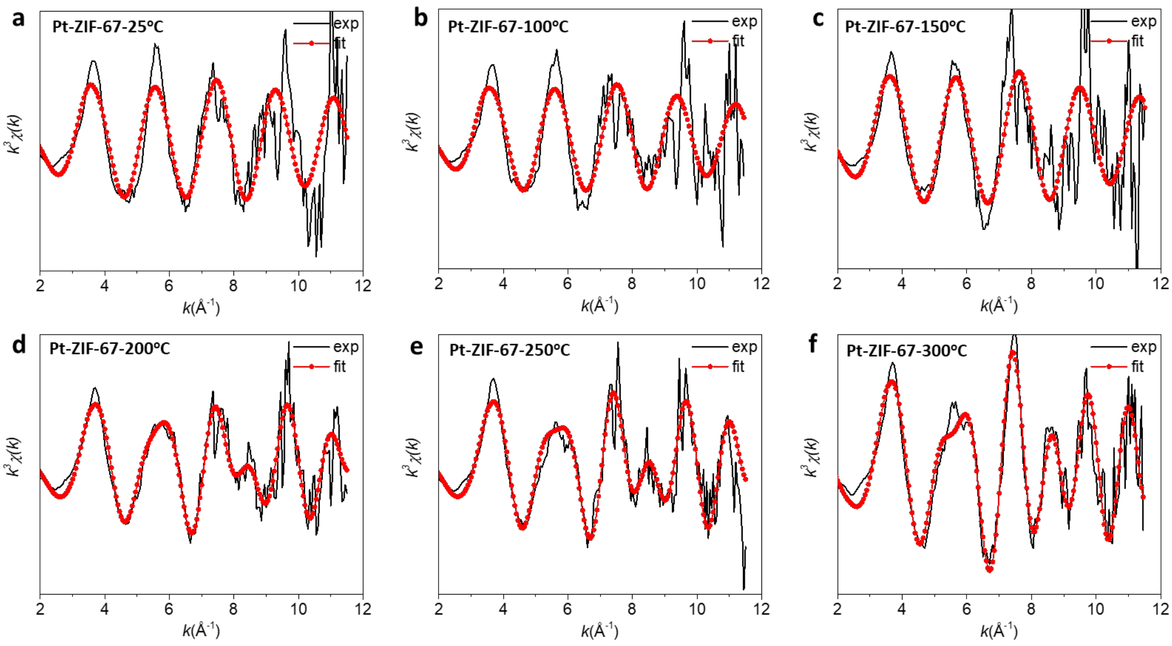

Fig. S4. In-situ Pt L_3-edge experimental (exp) and fitting (fit) EXAFS spectra of Pt-ZIF-67 catalyst under different pyrolysis temperatures.

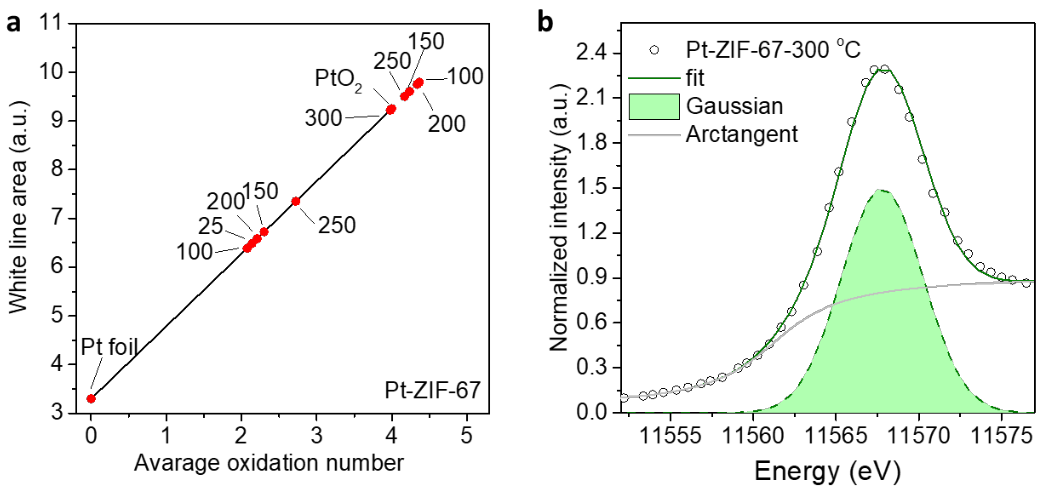

Fig. S5. (a) Relation between the average oxidation number and the white line area intensity of the Pt L_3-edge XANES of Pt-ZIF-67 under different pyrolysis temperatures. (b) Experimental determination of the white-line intensity at the Pt L_3 absorption edge in Pt-ZIF-67-300 °C. The open circle black line represents the normalized x-ray attenuation coefficient, μ(E), measured by XAS, while the solid black line represents the arctangent function used to model the continuum step at the L_3 absorption edge. The solid green line represents the best fit to the data using a Gaussian + arctangent fit function.

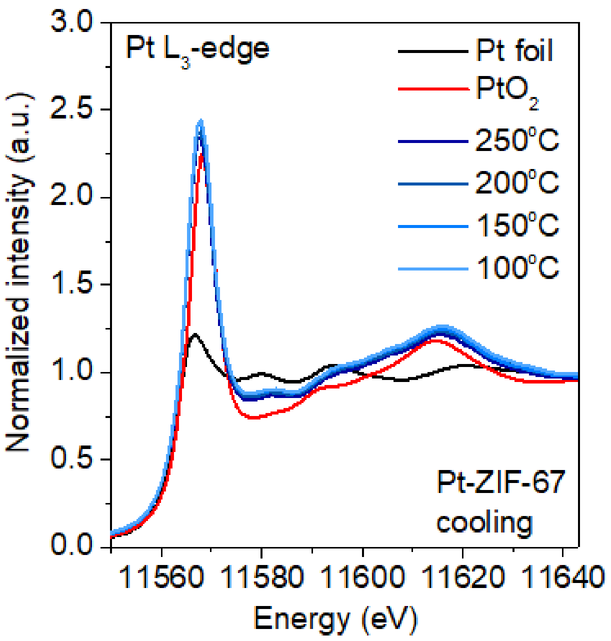

Fig. S6. In-situ Pt L_3-edge XANES spectra of Pt-ZIF-67 under different temperatures during cooling process.

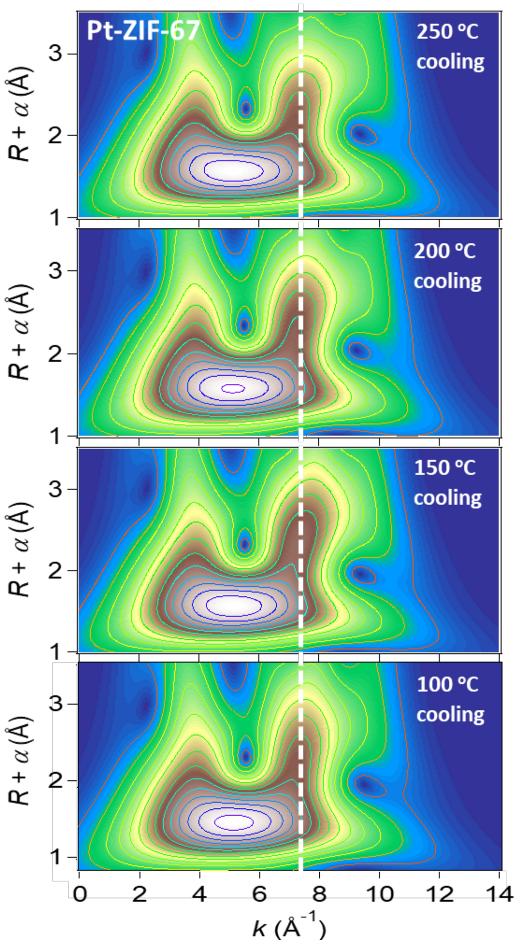

Fig. S7. In-situ Pt L_3-edge WT-EXAFS spectra of Pt-ZIF-67 under different temperatures during cooling process.

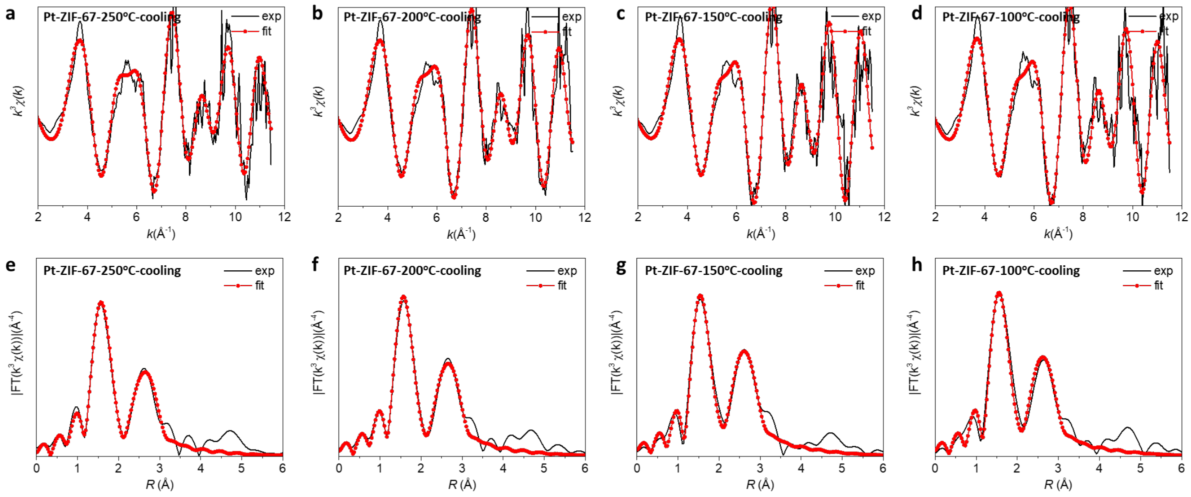

Fig. S8. (a-d) In-situ Pt L_3-edge experimental (exp) and fitting (fit) EXAFS spectra of Pt-ZIF-67 catalyst under different temperatures during cooling process. (e-h) Fourier-transformed magnitudes of in-situ Pt L_3-edge experimental (exp) and fitting (fit) EXAFS spectra of Pt-ZIF-67 catalyst under different temperatures during cooling process.

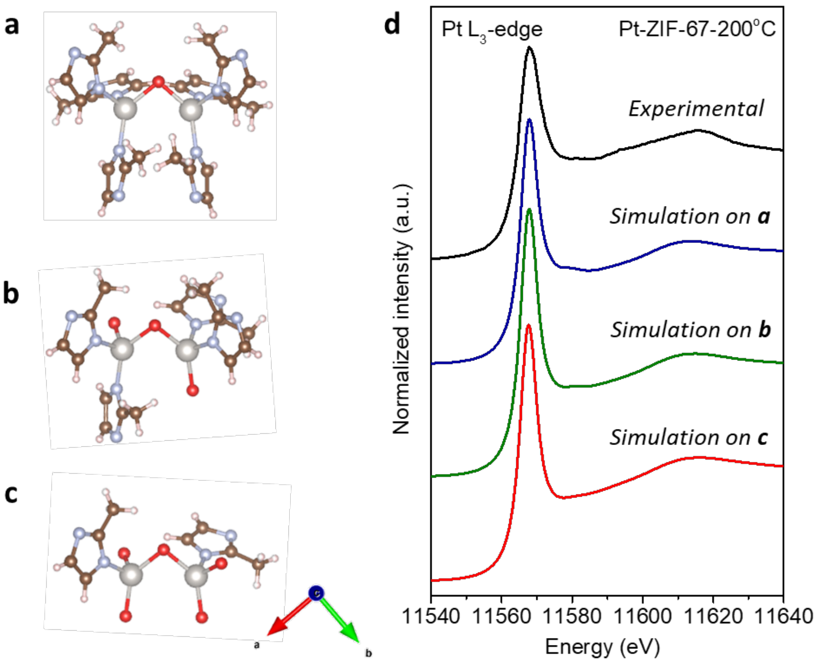

Fig. S9. (a-c) Representative structure models with the linking 2-methylimidazole rings between paired Pt single atoms partially replaced by oxygen atoms. (d) Comparison of experimental and simulated Pt L_3-edge XANES spectra of Pt-ZIF-67-200 °C achieved on different structure models.

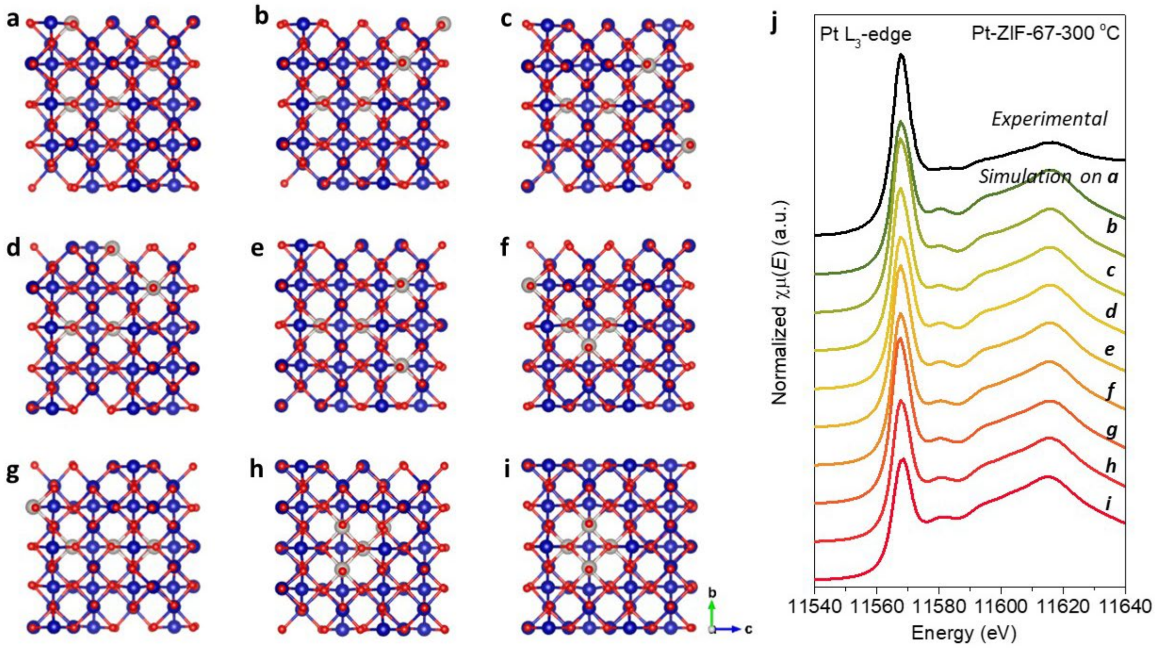

Fig. S10. (a-i) Representative structure models of Pt-Co_3O_4 with 4 Pt atoms incorporated in the lattice of Co_3O_4. Different number of Pt single atoms (2-4) group to exhibit Pt-Pt interactions. (j) Comparison of experimental and simulated Pt L_3-edge XANES spectra of Pt-ZIF-67-300 °C achieved on different structure models.

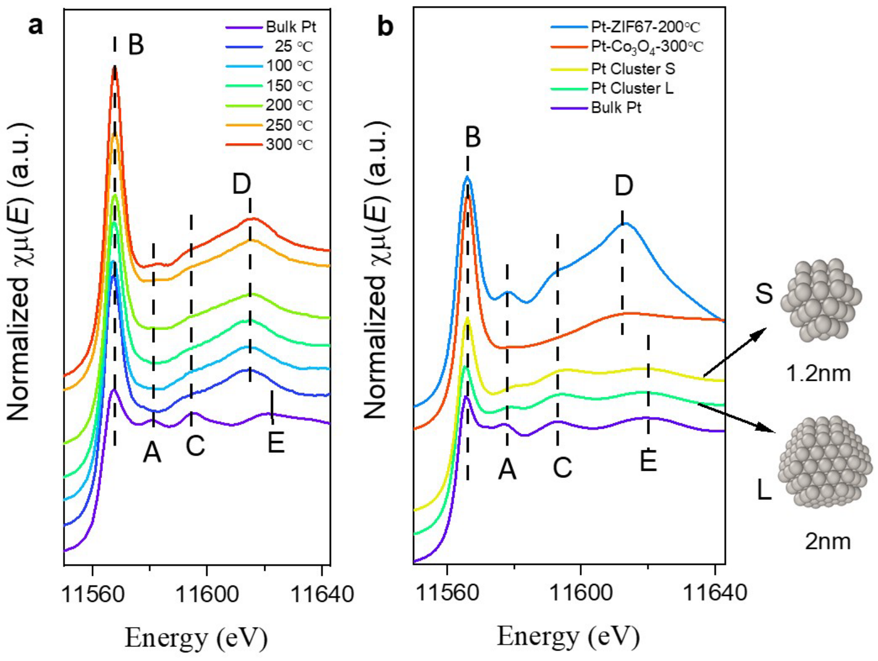

Fig. S11. (a) The experimental Pt-L_3 edge XANES spectra of Pt-ZIF-67 under different pyrolysis temperatures. (b) Comparison of the theoretical Pt-L_3 edge XANES spectra calculated based our proposed structure models at 200 °C and 300 °C and additional Pt cluster models. The experimental spectra for Pt-ZIF-67-200 °C and Pt-ZIF-67-300 °C show a pronounced main peak B at 11566 eV and three more feature peaks A, C and D at 11580, 11594 and 11614 eV, respectively. The theoretical spectra for Pt clusters show a much lower intensity for the main peak B and a new peak E at 11621 eV, and no feature at the energy position of 11614 eV for peak D was observed, which is in marked contrast the experimental spectra. Therefore, the possibility for direct Pt-Pt bonding in the Pt clusters or nanoparticles can be excluded.

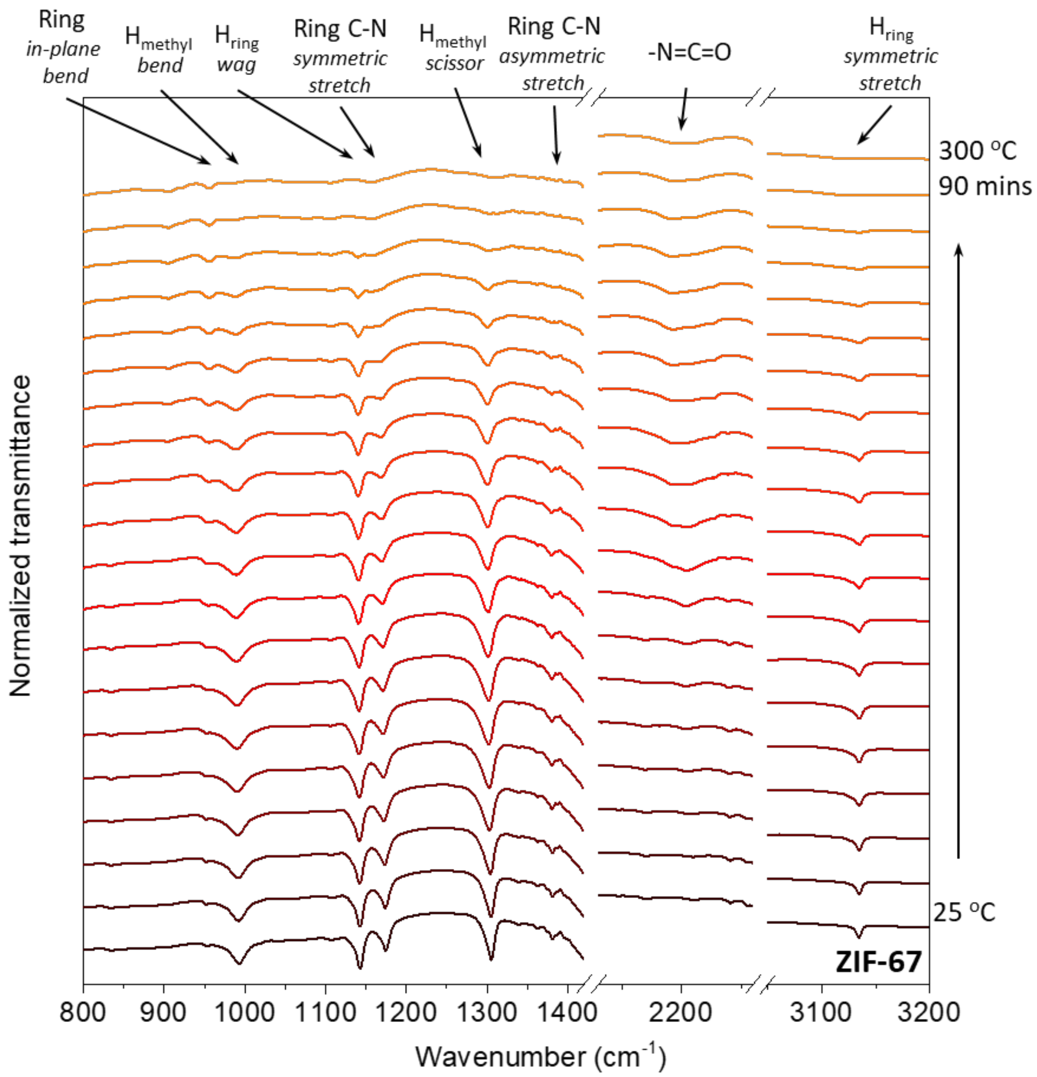

Fig. S12. In-situ temperature-dependent DRIFTs spectra of ZIF-67 with characteristic peaks indicated by arrows and labels.[14]

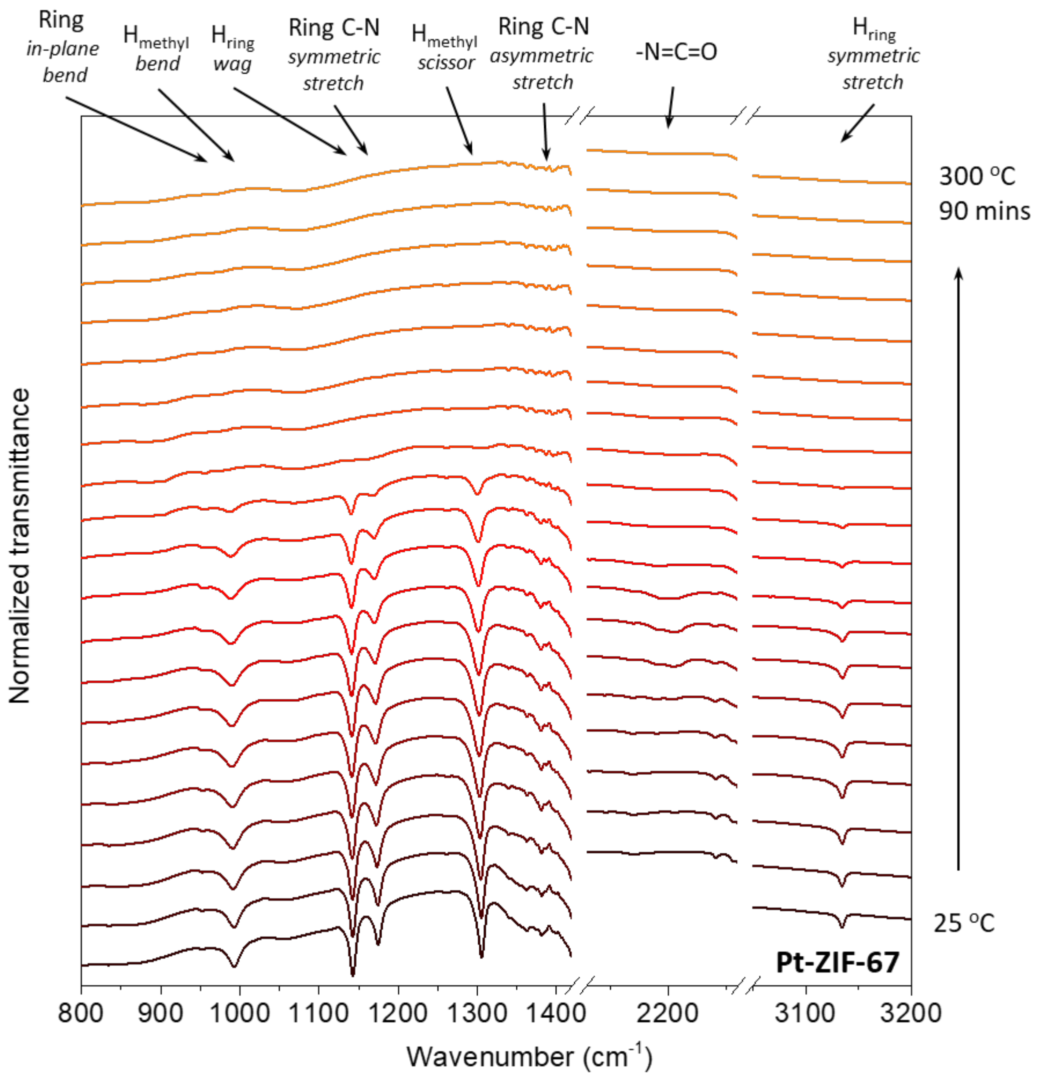

Fig. S13. In-situ temperature-dependent DRIFTs spectra of Pt-ZIF-67 with characteristic peaks indicated by arrows and labels.

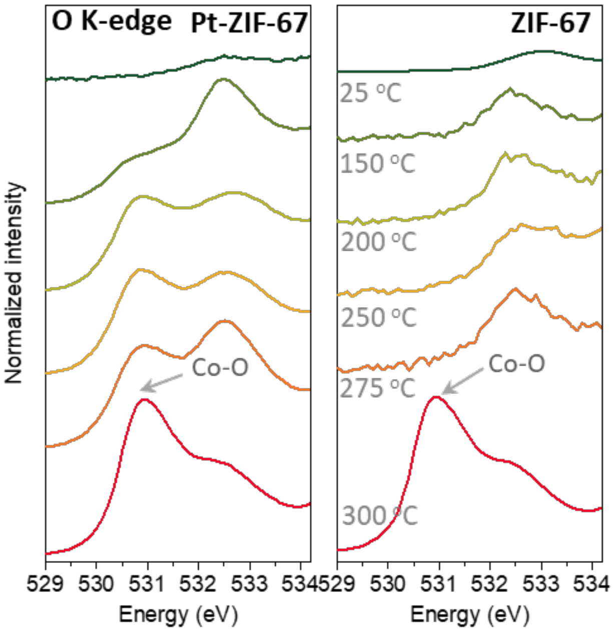

Fig. S14. Ex-situ O-K edge XANES spectra on Pt-ZIF-67 and ZIF-67 under different pyrolysis temperatures.

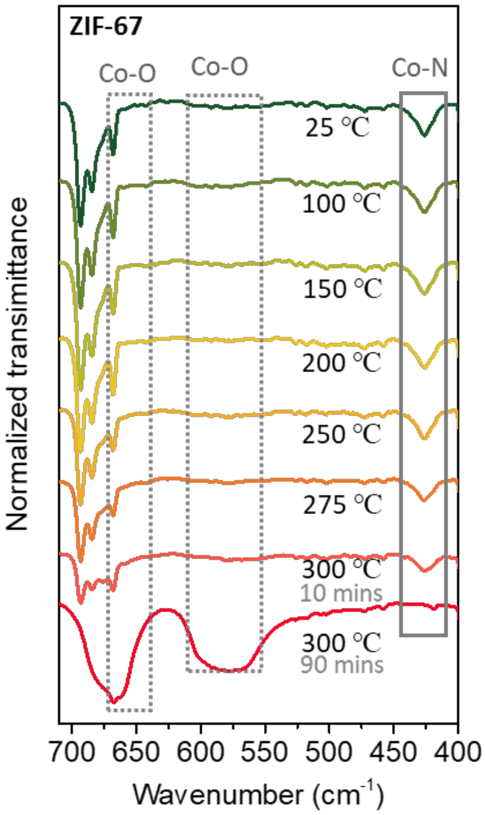

Fig. S15. Ex-situ DRIFTs spectra in low wavenumber region of ZIF-67 under different pyrolysis temperatures. The Co-N band is indicated by solid grey box and Co-O band is indicated by dotted grey box.

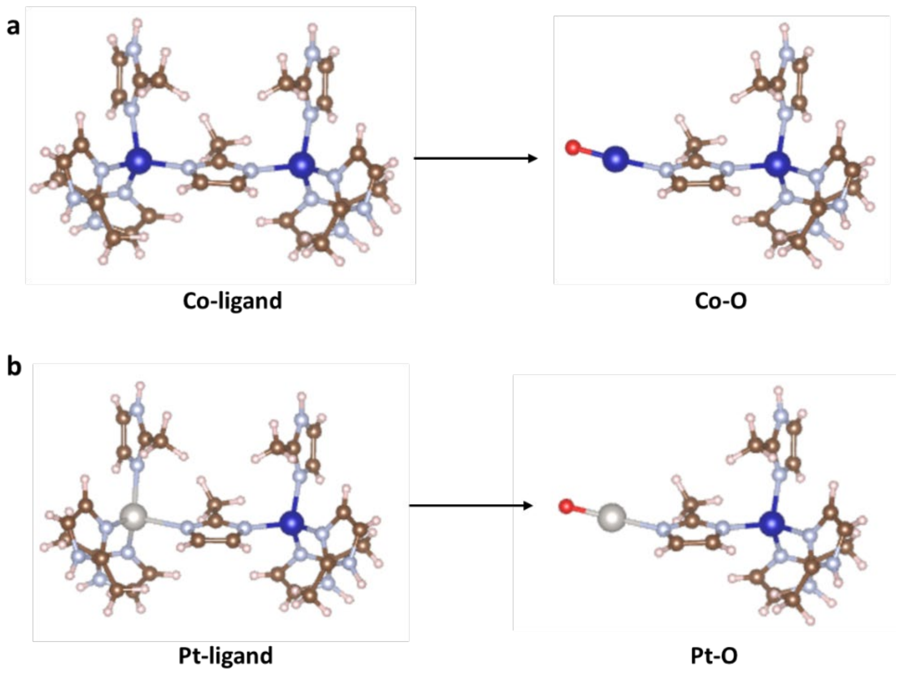

Fig. S16. DFT structure models of (a) Co-ligand to Co-O transition and (b) Pt-ligand to Pt-O transition.

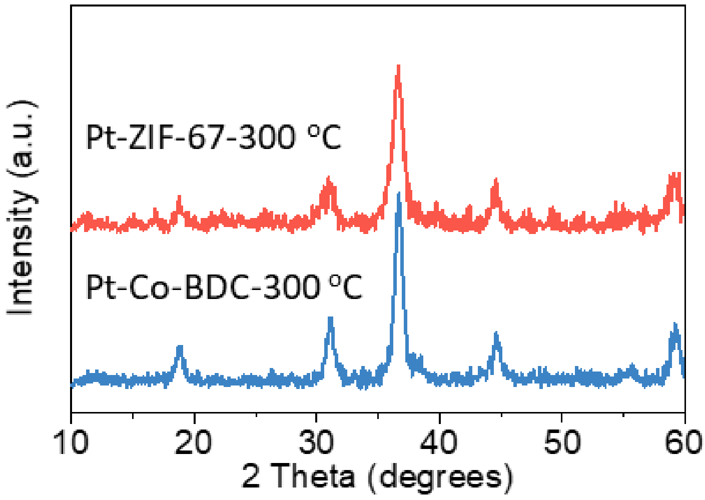

Fig. S17. XRD patterns of Pt-ZIF-67-300 °C and Pt-Co-BDC-300 °C hybrids, which demonstrate characteristic peaks corresponding to Co_3O_4 (PDF#: 42-1467) with no Pt-related peaks observed.

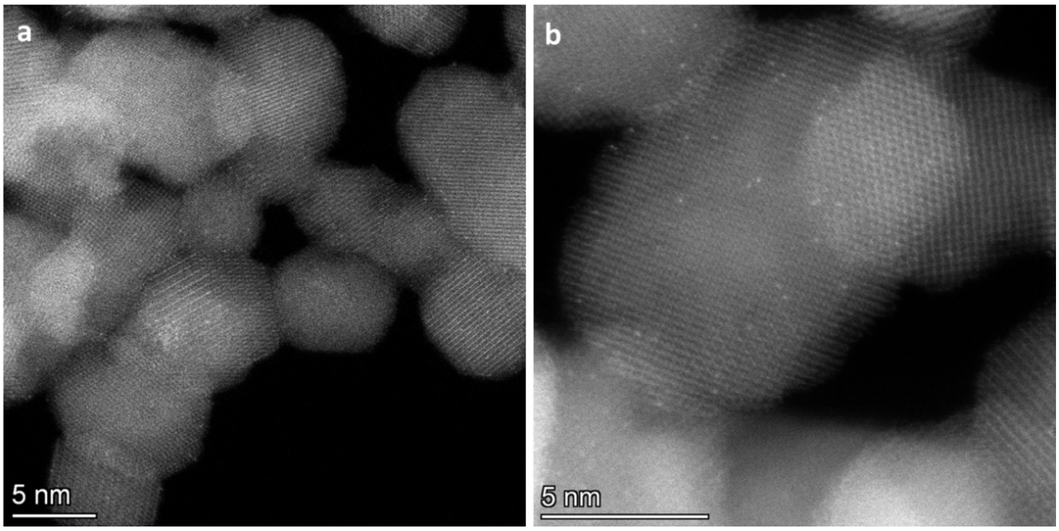

Fig. S18. HAADF-STEM images of Pt-Co-BDC-300 ºC.

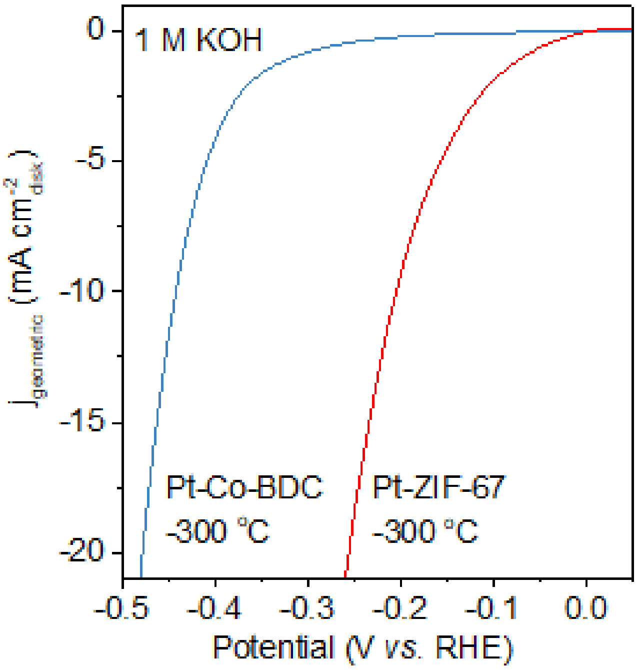

Fig. S19. Polarization curves of HER electrocatalysis normalized to surface area of disk electrodes in Ar-saturated 1 M KOH media for Pt-ZIF-67-300 ºC and Pt-Co-BDC-300 ºC catalysts.

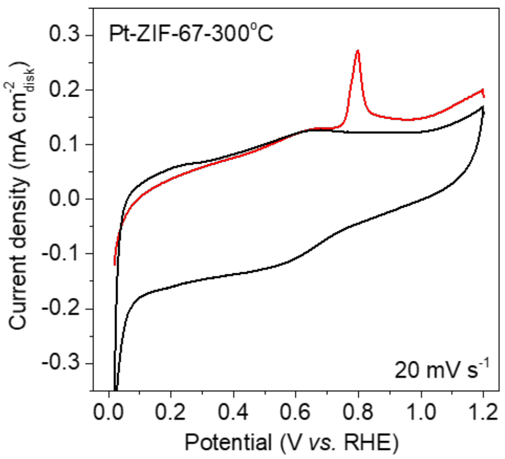

Fig. S20. CO stripping voltammograms of Pt-ZIF-67-300 °C and Pt-Co-BDC-300 °C in 0.1 M $HClO_4$ electrolyte with a scan rate of 20 mV s^{-1}. The sharp CO stripping peak of Pt-ZIF-67-300 °C derives from the high-density Pt single atoms, while in contrast, the Pt-Co-BDC-300 °C with low-density isolated Pt single atoms exhibits weak CO stripping peak.

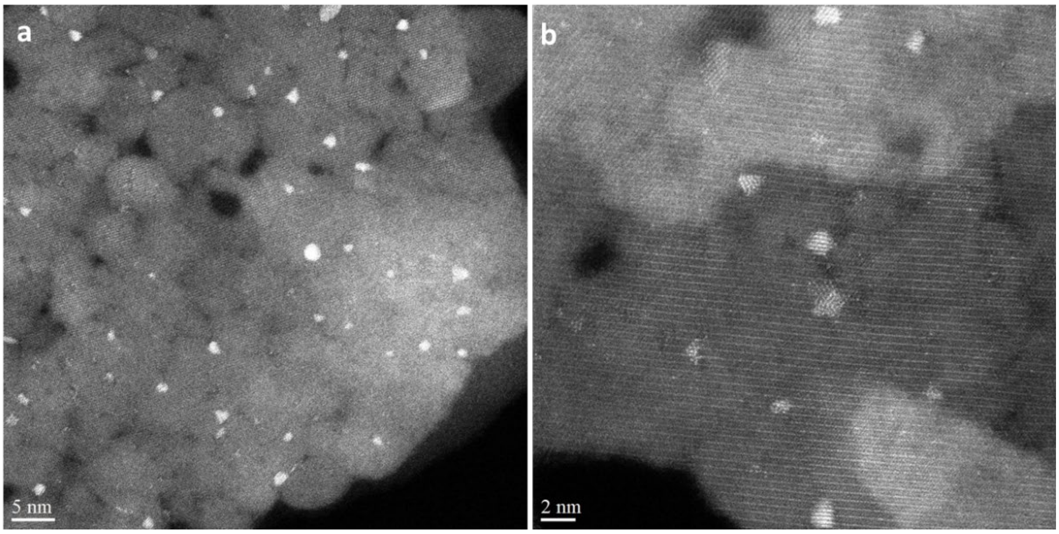

Fig. S21. HAADF-STEM images of Au-ZIF-67-300 °C.

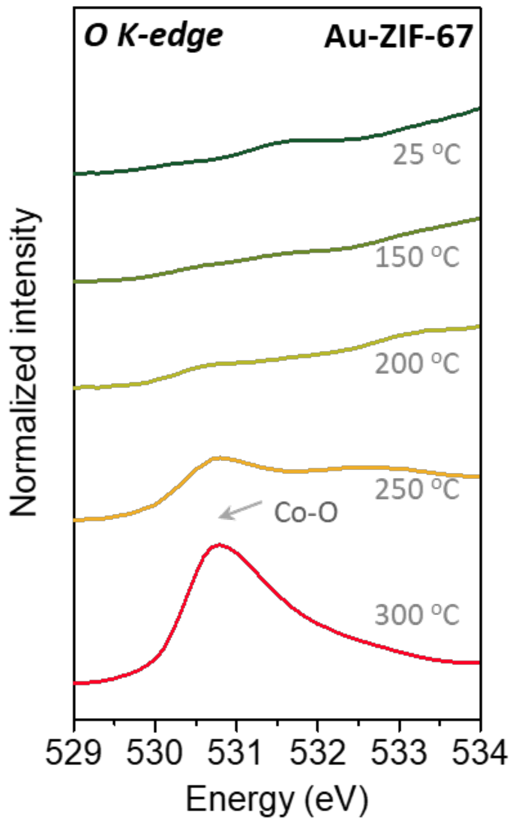

Fig. S22. Ex-situ O-K edge XANES spectra on Au-ZIF-67 under different pyrolysis temperatures, which indicate the formation of Co-O bond after 250 ºC.

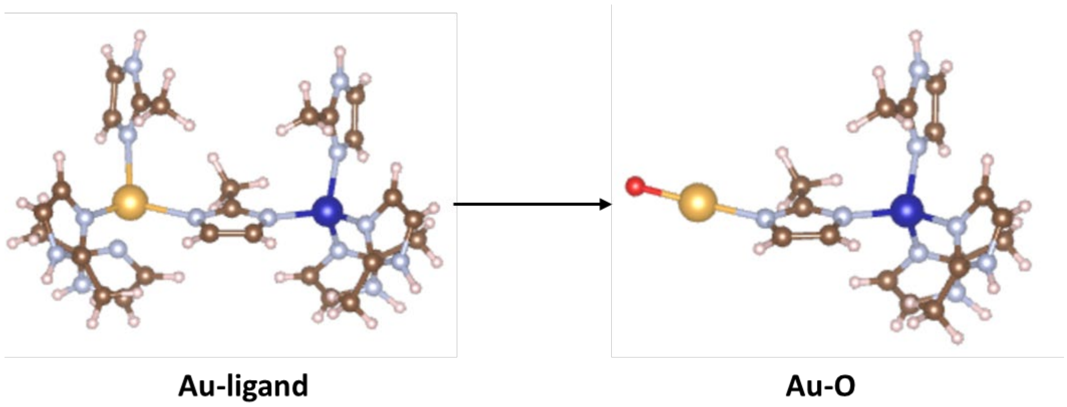

Fig. S23. DFT structure models of Au-ligand to Au-O transition.

Table S1. Pt L_3-edge EXAFS curve fitting parameters [a]

Sample	Path	*N*	***R*** (Å)	σ^2 ($Å^2$)
**Pt foil**	Pt-Pt	12	2.76	0.0044
**PtO_2**	Pt-O	6	2.01	0.003
**Pt-ZIF-67**	Pt-N	4.2	2.06	0.0021
**Pt-ZIF-67-100 °C**	Pt-N/O	4.1	2.04	0.0029
**Pt-ZIF-67-150 °C**	Pt-N/O	5.0	2.02	0.0030
**Pt-ZIF-67-200 °C**	Pt-N/O	4.2	2.01	0.0044
	Pt-Co	2.0	2.93	0.0093
	Pt-Pt	2.3	2.80	0.0072
**Pt-ZIF-67-250 °C**	Pt-N/O	4.3	2.03	0.0044
	Pt-Co	2.7	2.92	0.0094
	Pt-Pt	2.9	2.80	0.0073
**Pt-ZIF-67-300 °C**	Pt-O	6.4	2.03	0.0064
	Pt-Co	3.5	2.84	0.0046
	Pt-Pt	4.0	2.82	0.0036
**Pt-ZIF-67-250 °C (cooling)**	Pt-O	6.4	2.03	0.0046
	Pt-Co	3.5	2.85	0.0045
	Pt-Pt	4.0	2.83	0.0032
**Pt-ZIF-67-200 °C (cooling)**	Pt-O	6.4	2.03	0.0043
	Pt-Co	3.5	2.86	0.0045
	Pt-Pt	4.0	2.84	0.0029
**Pt-ZIF-67-150 °C (cooling)**	Pt-O	6.4	2.02	0.0042
	Pt-Co	3.5	2.85	0.0042
	Pt-Pt	4.0	2.81	0.0029
**Pt-ZIF-67-100 °C (cooling)**	Pt-O	6.4	2.02	0.0040
	Pt-Co	3.5	2.92	0.0040
	Pt-Pt	4.0	2.80	0.0027

[a] *N*, coordination number; *R*, distance between absorber and backscatter atoms; σ^2, Debye–Waller factor to account for both thermal and structural disorders; Error bounds (accuracies) that characterize the structural parameters obtained by EXAFS spectroscopy were estimated as $N \pm 20$ %; $R \pm 1$%; $\sigma^2 \pm 20$ %. Fitting range: $2.0 \leq k$ (/Å) ≤ 10.0 and $1.0 \leq R$ (Å) ≤ 3.0.

Table S2. Comparison of M/Co (M = Pt, Au) mass ratio, M weight % and M atom % in hybrid samples determined by ICP-MS.

Samples	Pt-ZIF-67	Pt-ZIF-67-300 °C	Pt-Co-BDC	Pt-Co-BDC-300 °C	Au-ZIF-67	Au-ZIF-67-300 °C
**M/Co mass ratio**	0.089	0.087	0.0042	0.0043	0.21	0.21
**M weight %**		5.95		0.31		13.32
**M atom %**		1.10		0.055		2.57

References:

[1] H. Hu, Bu Y. Guan, Xiong W. Lou, *Chem* **2016**, *1*, 102-113.

[2] S. Zhao, Y. Wang, J. Dong, C.-T. He, H. Yin, P. An, K. Zhao, X. Zhang, C. Gao, L. Zhang, J. Lv, J. Wang, J. Zhang, A. M. Khattak, N. A. Khan, Z. Wei, J. Zhang, S. Liu, H. Zhao, Z. Tang, *Nat. Energy* **2016**, *1*, 16184.

[3] C. Koch, Arizona State University **2002**.

[4] B. Ravel, M. Newville, *J. Synchrotron Radiat.* **2005**, *12*, 537-541.

[5] O. Bunau, Y. Joly, *J. Phys. Condens. Matter.* **2009**, *21*, 345501.

[6] a) G. Kresse, J. Furthmiiller, *Phys. Rev. B* **1996**, *54*, 11169-11186; b) G. Kresse, J. Furthmiiller, *Comp. Mater. Sci.* **1996**, *6*, 15-50.

[7] J. P. Perdew, K. Burke, M. Ernzerhof, *Phys. Rev. Lett.* **1996**, *77*, 3865-3868.

[8] P. E. Blöchl, *Physical Review B* **1994**, *50*, 17953-17979.

[9] a) J. Chen, X. Wu, A. Selloni, *Phys. Rev. B* **2011**, *83*, 245204; b) G. Lan, J. Song, Z. Yang, *J. Alloy. Compd.* **2018**, *749*, 909-925.

[10] K. A. Persson, B. Waldwick, P. Lazic, G. Ceder, *Physical Review B* **2012**, *85*, 235438.

[11] J. A. Dean, McGraw-Hill, New York, **1999**.

[12] J. Durst, C. Simon, F. Hasché, H. A. Gasteiger, *J. Electrochem. Soc.* **2014**, *162*, F190-F203.

[13] S. Rudi, C. Cui, L. Gan, P. Strasser, *Electrocatal.* **2014**, *5*, 408-418.

[14] a) T. Wang, Y. Wang, M. Sun, A. Hanif, H. Wu, Q. Gu, Y. S. Ok, D. C. W. Tsang, J. Li, J. Yu, J. Shang, *Chem. Sci.* **2020**, *11*, 6670-6681; b) C. Wu, D. Xie, Y. Mei, Z. Xiu, K. M. Poduska, D. Li, B. Xu, D. Sun, *Phys. Chem. Chem. Phys.* **2019**, *21*, 17571-17577.
